# The relationship between off-ice testing and on-ice performance in male youth Ice hockey players

**DOI:** 10.3389/fspor.2024.1418713

**Published:** 2024-08-15

**Authors:** Mark S. Rice, Darren E. R. Warburton, Alejandro Gaytan-Gonzalez, Veronica K. Jamnik, Kai Kaufman, Declan R. D. Warburton, Michael Souster, Shannon S. D. Bredin

**Affiliations:** ^1^ Cognitive and Motor Learning (LEARN) Laboratory, Faculty of Education, University of British Columbia, Vancouver, BC, Canada; ^2^ School of Kinesiology, University of the Fraser Valley, Chilliwack, BC, Canada; ^3^ Physical Activity Promotion and Chronic Disease Prevention Unit, Faculty of Education, University of British Columbia, Vancouver, BC, Canada; ^4^ Experimental Medicine Program, Faculty of Medicine, University of British Columbia, Vancouver, BC, Canada; ^5^ School of Kinesiology and Health Science, York University, Toronto, ON, Canada; ^6^ Health & Fitness Society of BC, Vancouver, BC, Canada

**Keywords:** off-ice testing, on-ice performance, ice hockey, youth players, predictive metrics

## Abstract

**Introduction:**

Ice hockey demands a unique blend of physical fitness and skill, necessitating a comprehensive understanding of the factors influencing on-ice performance. The present study was designed to examine the relationship between off-ice and on-ice performance measures in male, youth, ice hockey players.

**Methods:**

Eleven minor hockey players (Age = 9.8 ± 1.1 years) participated in two testing days: (1) off-ice and (2) on-ice assessments. Off-ice assessments included maximal aerobic power, anaerobic fitness, muscular strength (handgrip and single leg squat), muscular endurance (curl-ups and push-ups), muscular power (standing long jump and vertical jump), and 30 m sprinting speed and acceleration. On-ice testing included a 15.2 m maximum speed test, a 6.1 m acceleration test with a continuation into a 47.9 m top speed test, an agility cornering S turn test, and a shot velocity test.

**Results:**

Twenty-four out of 33 off-ice variables were significantly correlated with at least one of the 11 on-ice performance variables. From those 24, 10 were included as predictors for at least one of the on-ice performance variables. Each model was composed of either one or two predictors, where the most common predictors were 30 m Run – Split (6.1 m) Sprint time and 30 m Run – Total (30 m) Sprint Max speed (included in four out of 11 models each). The prediction formulas R2 and coefficient of variation ranged from 0.63% to 0.96% and 1.2% to 15.3%, respectively.

**Discussion:**

Diverse off-ice measures of aerobic fitness, anaerobic power, muscular strength, power, and endurance, and sprinting speed, acceleration, and agility are predictive of on-ice performance. The insights gained from this study contribute to the refinement of assessment protocols, fostering a comprehensive approach to optimizing player performance and potential. Understanding the connection between objective off-ice testing and on-ice performance can support tailored training programs and player development in male youth ice hockey.

## 1 Introduction

Ice hockey, renowned for its dynamic and fast-paced nature, demands a unique set of physical attributes and skills (1–3). As the sport evolves, understanding the factors that contribute to on-ice success becomes increasingly important for the development of the athlete. Ice hockey is characterized by a combination of speed, acceleration, strength, power, agility, endurance, and skill (2, 4–6). While traditional on-ice metrics have long been the cornerstone of player evaluation, the role of off-ice testing in predicting on-ice success remains an intriguing avenue of exploration. Off-ice testing, encompassing a range of physical assessments, aims to quantify an athlete's fitness and conditioning – elements that are believed to correlate with on-ice performance (4, 6). This information can be invaluable for athletes in their training, allowing them to identify specific areas for improvement and tailor their conditioning programs, with the goal of enhancing their on-ice capabilities. Researchers have increasingly correlated off-ice fitness measures with on-ice performance. For instance, pioneers in this field, Drs. Norman Gledhill and Veronica Jamnik (York University), who were responsible for developing the National Hockey League (NHL) draft combine (7) and through a series of related publications, have revealed the importance of off-ice fitness measures for optimal performance and/or career progress in elite hockey players (including entry draft and rostered players in the NHL) (1, 4, 8–11). A recent systematic review of the literature (2) revealed that most of the research in this field has examined the on-ice performance of four major components: aerobic capacity, acceleration-speed, agility and change in direction, and repeated sprint ability. These tests were designed for wide-ranging purposes, such as assessing on-ice physiological responses and fitness levels, identifying talent and team selection, evaluating the effectiveness of training interventions, and for validation.

The predominant body of research in this area focuses on older cohorts (≥16 years), which suggests that there is a notable gap in the literature regarding young athletes. Bournival and colleagues (2) revealed that young athletes under the age of 12 years are rarely evaluated. Yet, participation in a sport like ice hockey begins in the early years in a number of countries globally. For example, ice hockey is often revered as a part of Canada's national identity and holds cultural significance. Structured programming begins for skill development starting in early childhood, typically around 4–6 years of age. Understanding the performance of young athletes in both on-ice and off-ice protocols is important for optimizing an athlete's developmental trajectory, but also for long-term health-related physical fitness and wellness.

Team selection decisions at the youth level overwhelmingly rely on the on-ice evaluation of players by coaches, volunteer observers, and parents. This subjective assessment of skill is influenced by the observers' own experience and training (12). Recent evidence indicates that the evaluation of off-ice performance may be even more important for the prediction of on-ice performance in younger populations owing to differences in maturation and the impact of physical attributes on performance (vs. experience-related technique) at this developmental age (13). We believe that the evaluation of both on-ice and off-ice performance indicators provides valuable information for coaches, athletes, and parents alike. For example, implementing another evaluation protocol in minor hockey, such as objective off-ice testing, could help identify those players who are physically fit, strong, powerful, or a combination of all three, which might be otherwise hidden when using subjective on-ice evaluation in isolation.

Off-ice assessments are generally simple and easy to measure, making them highly feasible for youth cohorts. Many of the tests are familiar to young athletes through participation in other standardized physical fitness batteries, such as those administered as part of physical education in the school setting (e.g., push-ups, standing long-jump). Implementing off-ice protocols can help address limitations in the evaluation and training of young athletes related to the availability and economic costs associated with ice rentals. Moreover, these assessments can provide a comprehensive understanding of an athlete's physical attributes and areas for growth, facilitating the development of personalized training regimens to optimize both player performance and physical fitness. For instance, recently there has been an increased appreciation of the importance of sport specific testing for youth and adult athletes alike in a variety of sports. Sport-specific testing protocols have been created for wide-ranging sports (e.g., https://www.sporttesting.com) to help identify strengths and weaknesses of athletes. Accordingly, this study examined the complex relationships between age-appropriate off-ice testing measures and on-ice performance among male youth ice hockey players. This study aimed to bridge the gap between off-ice testing outcomes and the diverse challenges faced on the ice, specifically addressing the lack of information about male ice hockey players under the age of 12 in the existing literature. The primary purpose of this investigation was to examine the relationship between off-ice performance measures and sport-related performance during on-ice assessments in young male ice hockey players. We hypothesized that players who exhibit higher scores on off-ice measures would also demonstrate better scores on on-ice measures. It was also postulated that off-ice measures requiring high power output from the legs (such as vertical jump, standing long jump, the Wingate cycle ergometer test, and the 30-m sprint) would be particularly important in predicting on-ice performance (such as skating speed, acceleration, and agility).

## 2 Methodology

### 2.1 Participants

Fourteen male minor hockey players were recruited across three birth years (2004, 2005, and 2006). Complete data was available from 11 participants. Approximately 45% (5 of 11) of the recruited players (e.g., those born in 2004–2006) have gone onto to play Major Junior or Junior A hockey. The sample ranged in age from 9 to 11 years. All participants were pre-screened for physical activity by completing the Physical Activity Readiness Questionnaire for Everyone (PAR-Q+) (14). No individual was excluded from participating based on pre-screening (i.e., all participants responded “NO” to each question on page 1 of the PAR-Q+). The investigation received approval from and was executed in exact accordance with the ethical guidelines set forth by the University of British Columbia's Behavioural Research Ethics Board (H14-03174) for research involving human participants. All participants' legal parent and/or guardian provided written informed consent and all players provided informed written assent in accordance with the Declaration of Helsinki. There were no incidents of injury during the on-ice and off-ice testing sessions.

### 2.2 Procedures

Participants were asked to volunteer for testing on two separate days, with a total time commitment of 3 h (90 min per day). Players performed a battery of 14 off-ice tests and four on-ice tests (15).

On-ice assessments were conducted at a local ice hockey arena, and all off-ice assessments were administered at the Cognitive and Motor Learning Laboratory, Physical Activity Promotion and Chronic Disease Prevention Unit at the University of British Columbia.

Off-ice assessments included measurements of anthropometrics, muscular strength, muscular endurance, muscular power, flexibility, anaerobic fitness, aerobic fitness, speed, acceleration, and agility. On-ice assessments included measurements of skating speed, acceleration, agility, and shot velocity.

### 2.3 Day 1: questionnaires and off-ice assessments

**Figure 1:**
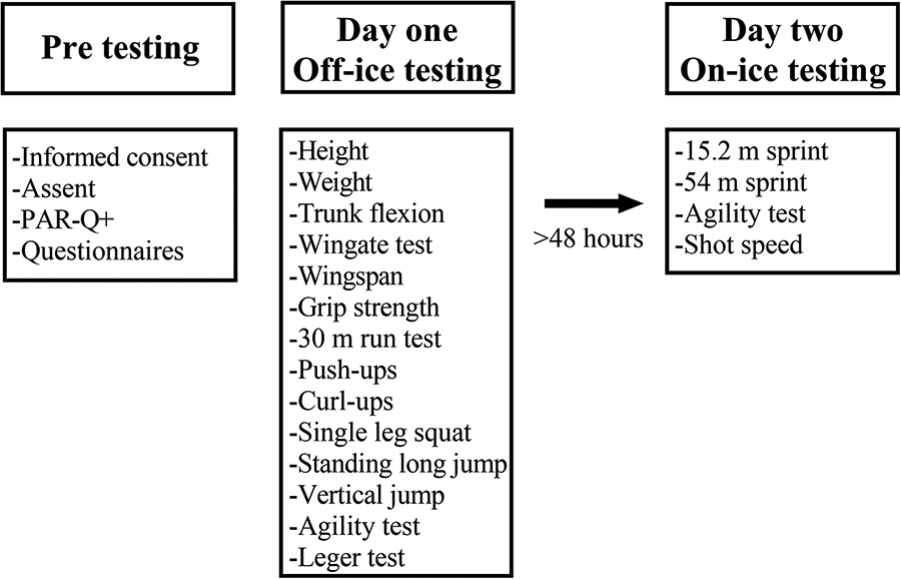
Schematic representation of the investigation procedure.

Each participant completed the PAR-Q+ and a demographic questionnaire, which included playing experience. Participants were then tested in the same order for each test including height, weight, sit and reach flexibility, Wingate anaerobic test, Wingspan, handgrip strength, 30 m sprint, push-ups, curl-ups, one-legged squat, standing long jump, vertical jump, pro-agility test, and the Leger 20 m shuttle run (Figure 1). The order of tests was conducted in the same way for each participant, ensuring adequate rest time between intense tests to allow for appropriate recovery. The order of testing was designed to mimic that currently done in combine testing at the developmental and elite levels.

### 2.4 Day 2: on-ice assessments

The on-ice assessments required the participants to be dressed in their complete hockey apparel, including helmet and all protective gear. Participants were then led through a standardized warm-up including two laps around the ice at a minimal pace, two laps around the ice at a −60% pace, and two laps around the ice at an −80% pace. Participants were then tested in the same order for each test including: 15.2 m maximal speed test, 6.1 m acceleration to 47.9 m full speed test, shot velocity, and agility test.

### 2.5 Assessments

Players performed a battery of 14 off-ice tests and four on-ice tests (15). Off-ice assessments included measurements of anthropometrics, muscular strength, muscular endurance, muscular power, flexibility, anaerobic fitness, aerobic fitness, speed, acceleration, and agility. On-ice assessments included measurements of skating velocity, skating acceleration, skating agility, and shot velocity.

#### 2.5.1 Off-ice assessments

##### 2.5.1.1 Anthropometry

Anthropometric assessments included non-invasive evaluations of standing height, Wingspan, and body mass. Standing height was measured (to the nearest cm) with a Seca stadiometer (Birmingham, United Kingdom). Participants stood without footwear and with heels together. Wingspan, measured to the nearest cm, involved participants extending their arms at shoulder height with their back against the wall. Body weight, measured to the nearest 0.1 kg, was assessed using a Tanita digital scale (Tanita TBF-300WA, Arlington Heights, Illinois, USA). During all anthropometric measurements, participants dressed in shorts and t-shirts and without socks and footwear.

##### 2.5.1.2 Musculoskeletal fitness

###### 2.5.1.2.1 Muscular strength

Hand grip strength was measured using an Almedic Hand Grip dynamometer, with participants squeezing the dynamometer forcefully at a 45° angle from the body. Two trials for each hand were conducted, recording the maximum force (kg) achieved.

The single leg squat was used in the overall assessment of musculoskeletal fitness by providing information on the participants' lower body strength, balance, and control. Single leg squat performance was assessed using a point system, with participants performing five consecutive repetitions on each leg, resulting in a total possible score of 150 points (7). Therefore, each squat was worth 15 points with a maximum score of 75 per leg. This test was previously included in the NHL combine testing (7). Participants began with one foot on the ground (the scoring leg) and the other leg hanging freely beside it. The movement started with a slow descent until the top of the participant's thigh was parallel to the ground. As the participant descended, the hanging leg and arms would rise forward and upward, reaching a straight position in front of them, parallel to the ground. The gluteal muscles were not allowed to come in contact with the heel during both the downward and upward phases of the movement. There was a 1-s pause required at both the bottom and top of the movement. Throughout the entire movement, the participant's spine was to remain extended, ensuring an upright posture.

###### 2.5.1.2.2 Muscular endurance

Curl-ups were performed to a metronome set at 25 beats per minute, with participants completing as many as possible within 4 min (i.e., to a maximum of 100). The participant began in the supine position, knees bent at an angle of 90°, heels in contact with the floor, and arms crossed over the chest. Push-ups were executed at a rate of 25 beats per minute, with proper form and a 90° elbow angle. Each test was terminated under standardized conditions, including discomfort, inability to maintain cadence, or failure to maintain proper technique. All tests were conducted according to the standards established by Gledhill and Jamnik for the NHL entry draft combine (7).

###### 2.5.1.2.3 Muscular power

Standing (horizontal) long jump was measured using the distance jumped from a standing position, with participants given two attempts, and the highest score recorded (cm). Vertical jump (cm) was assessed using the Vertec apparatus (Vertec Power Systems, Knoxville, Tennessee, USA), with participants jumping as high as possible.

###### 2.5.1.2.4 Flexibility

Trunk forward flexion (sit and reach test) was measured via standardized procedures (15) to the nearest 0.5 cm using a flexometer with the bottom of the foot set at the 25.5 cm mark of the measuring scale (Warburton Flexometer, Vancouver, BC).

##### 2.5.1.3 Agility

Off-ice agility was assessed using the pro agility (5-10-5) test and Brower TC timing gates with a precision of 0.01 s. Participants, positioned 10 cm behind the start line, faced a consistent direction, and their initial movement determined the recorded direction. Starting from this position, participants sprinted 4.6 m (15 ft) to the right, touched the line with their right hand, swiftly changed direction, sprinted 9.1 m (30 ft) to the left, touched the line with their left hand, changed direction again, and sprinted 4.6 m (15 ft) back to the start/finish line. The timing started when passing through the gates on the start/finish line and stopped on the second pass. The split time was determined when the participant passed by the start/finish line after changing directions the first time. Each participant performed the test twice in each direction (i.e., right and left), with the fastest time recorded. Additional attempts were allowed for stumbling, falling, or slipping.

##### 2.5.1.4 Anaerobic fitness

The 30-s Wingate cycle ergometer test and the 30-m sprint test were completed to measure anaerobic fitness.

###### 2.5.1.4.1 30-s Wingate

**Figure 2:**
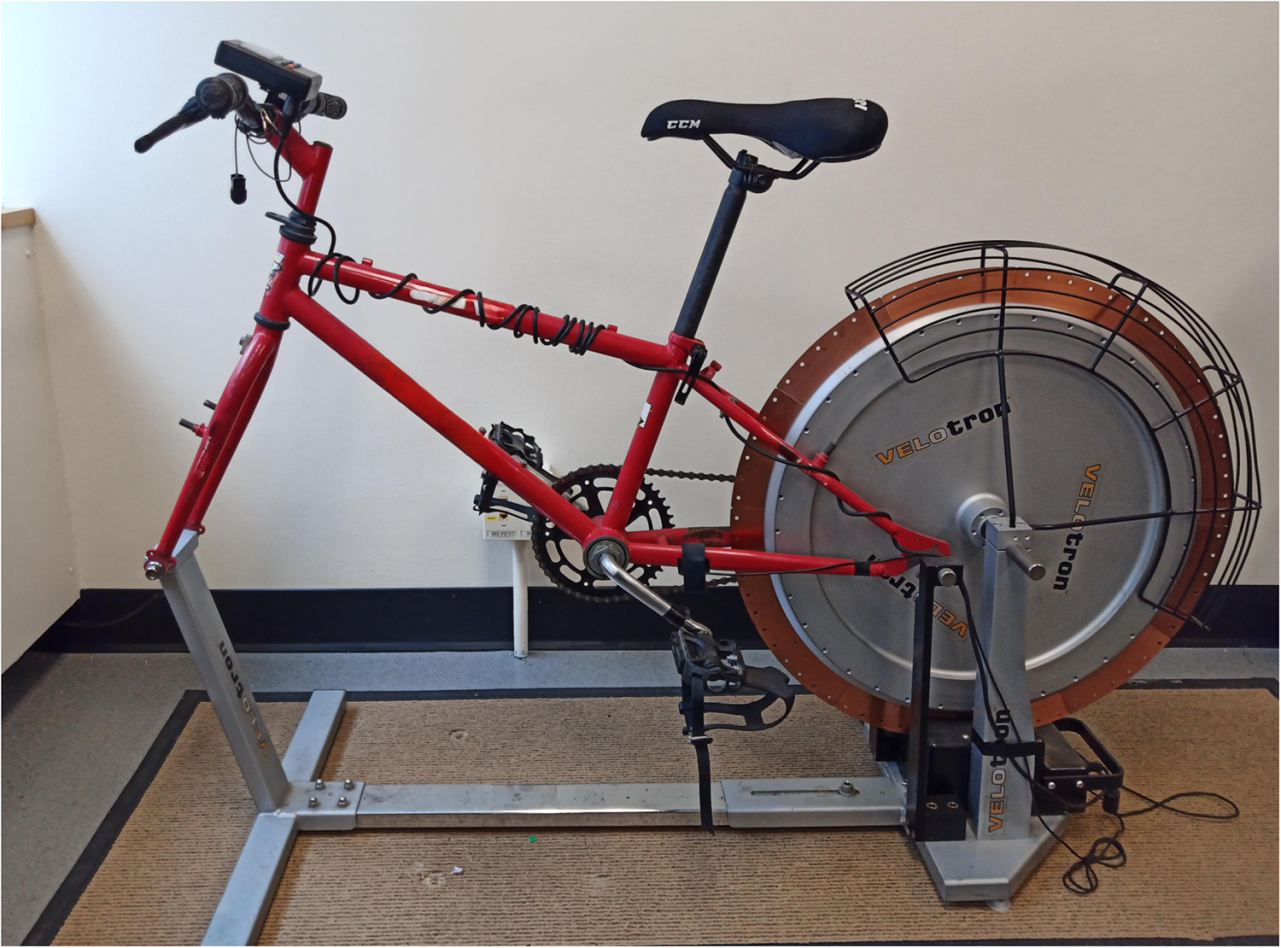
Electronic bike used for 30-s Wingate cycle ergometer test as modified for youth participants.

A Racermate Velotron cycle system was modified with a youth bike frame to accommodate the younger participants (Figure 2). Participants were fitted to ensure a slight leg flexion when pedals were down and underwent a 2 min warm-up. Starting with a 10 s countdown, participants pedaled progressively faster until reaching maximum speed whereupon the calculated resistance (7.5% of body weight) was applied. The resistance applied differed from the NHL combine assessments (9.0% of body weight resistance) (7) to 7.5%, which is believed to be a more optimal load for a younger age cohort (16) and has been shown to be a reliable and valid means to measure anaerobic power (17). The Wingate test lasted 30 s against the designated resistance, followed by a 1 min cool-down. Recorded data (via the dedicated Velotron Wingate software) included anaerobic power output (W·kg^−1^), peak power output (W), mean power output (W), minimum power output (W), and fatigue index (in W·s^−1^ and %).

###### 2.5.1.4.2 30-m sprint

The 30-m sprint was used to measure anaerobic sprinting ability. The 30-m sprint was measured (via Brower TC timing gates) to the nearest 0.01 s including a 6.1-m split time. Participants performed the test twice with the fastest time being recorded as their score. If the participants were to stumble, fall, or slip, they were given another attempt.

##### 2.5.1.5 Aerobic fitness

Aerobic fitness was assessed using the 20-m multistage shuttle run test (18). The final stage reached was used to calculate maximal aerobic power (VO_2_max) using the Leger equation for children (18):

$$\begin{array}{r} VO_{2}\max\;(\mathrm{ml}\cdot kg^{-1}\cdot \mathrm{mi}n^{-1})=\;31.025+(3.238\times \mathrm{Speed}\;[\mathrm{km}\cdot h^{-1}])\\ -(3.248\times \mathrm{Age}\;[y])\\ +(0.1536\times \mathrm{Speed}\;[\mathrm{km}\cdot h^{-1}]\times \mathrm{Age}\;[y]) \end{array}$$

#### 2.5.2 On-ice assessments (full equipment)

##### 2.5.2.1 15.2 m maximal speed test

The on-ice maximal speed test was measured to the nearest 0.01 s (Brower TC timing gates). Participants started from the opposite blue line and began skating toward the defensive end of the ice. The participant would then circle around the defensive zone face-off dots picking up speed as they headed toward the defensive zone blue line. The first set of timing gates were set up on the defensive zone blue line at which time, the participant was at full skating speed when they broke the plane of the first timing gate. The participant then skated as fast as they could for 15.2 m to the offensive zone blue line where another set of timing gates were set up to measure the total amount of time. Participants performed the test twice with the fastest time being recorded as their score. If the participant were to stumble, fall, or slip, they were given another attempt.

##### 2.5.2.2 On-ice 54 m test

The on-ice 54-m test (including a 6.1 m acceleration test with a continuation into a 47.9 m top speed test) was measured to the nearest 0.01 s including a 6.1-m split time measured to the nearest 0.01 s using Brower TC timing gates. Participants started 10 cm behind the starting line in a ready position. The tester would countdown from three before saying, “go”. On the word “go”, the participant would skate as fast as they could for 54 m (i.e., from corner to corner of the rink). The timing would start when the participant passed through the first timing gate and stop when they passed through the last timing gate 54 m away. There was another timing gate 6.1 m from the start line to measure the participant's acceleration time. Participants performed the test twice with the fastest time being recorded as their score. If the participant were to stumble, fall, or slip, they were given another attempt.

##### 2.5.2.3 Agility cornering S turn test

On-ice agility was measured to the nearest 0.01 s using Brower TC timing gates. Participants would start from a ready position in the center of the goal crease in one zone of the rink 10 cm behind the starting timing gate. The participants always faced the same way to start the test, and depending on what direction they started the test, that was the direction that was recorded (i.e., if the first movement was to the right, the recorded score was to the right). The participant would then skate in one direction around the big faceoff circle and continue on to the other big faceoff circle making a large “S” shape. Once around the second faceoff circle, the participant skated as fast as possible to the blue line (18.9 m away from the icing line) until they broke the plane of the second timing gate. Participants performed the test twice in each direction with the fastest time recorded as their score for each direction. If the participant were to stumble, fall, or slip, they were given another attempt.

##### 2.5.2.4 Shot velocity test

Shot velocity was measured to the nearest mile per hour using a Bushnell Radar Gun (Bushnell, Vaughan, Ontario, Canada) from a distance of 9.1 m (30 ft). The participants shot the puck as hard as they could towards the net. Participants were given the choice to use whichever shot they felt had more velocity. Participants were allowed to skate into the shot; however, the puck had to remain 30 ft from the net when contacted. Participants were allowed two attempts to record the fastest score. Participants were given another attempt if they missed the net, broke a stick, stumbled or fell, or the radar gun did not register the shot.

### 2.6 Data calculations

The average speed (m·s^−1^) was calculated from the 30 m running (split and total), on-ice 15 m sprint, and on-ice 54 m sprint (split and total) tests by dividing the covered distance by the time spent to complete such a distance. The maximal sprinting speed (m·s^−1^) and relative acceleration (tau; expressed in s) were calculated by using non-linear least squares regression with the *shorts* package (version 3.0.0) in RStudio (version 2023.12.0 + 369; R version 4.3.2) for Windows (19). The data originally recorded in imperial units for vertical jump and shot speed (i.e., inches, and miles per hour) were transformed into international system units (i.e., cm, and km·h^−1^).

### 2.7 Statistical analyses

All variables were analyzed for normal distribution using the Shapiro-Wilk test. Almost all variables met the assumption of normality, except the 54 m skating tau (Shapiro-Wilk *p*-value = 0.012). Descriptive statistics included means, standard deviations (SD), and minimum and maximum scores. The first analysis consisted of bivariate correlations between off-ice and on-ice performance variables using the Pearson correlation coefficient (r). We then used linear regression analyses to develop simple prediction formulas from each significant off-ice performance variable to predict on-ice performance variables. Finally, we included all the significantly correlated variables into one model per on-ice performance variable using the forward method (to include the most relevant predictors). The variance inflation factor (VIF) was calculated for the models with two or more predictors and inspected to determine if the predictors on the final models showed high collinearity. If the forward method picked one variable with a VIF > 5 (20), we discarded that variable from the model and re-ran the analysis. We reported the statistical significance (*p*-value), coefficient of determination (R^2^), standard error of estimation (SEE; also known as standard deviation of the residuals), coefficient of variation (CV), and VIF (if applicable) for the developed prediction models. The CV was calculated as follows:

$$\mathrm{CV}(\text{\%})=\left(\frac{SEEpm}{Mean\;Oy}\right)\;\times \;100$$

Where *SEEpm* is the standard error of the estimation from one specific prediction model, and *Mean Oy* is the mean of the outcome on-ice variable the model attempts to predict.

All analyses were performed using IBM SPSS Statistics version 29.0.1 for Windows (IBM Corp., Armonk, NY, USA), whereas the graphs were drawn on GraphPad Prism version 10.1.2 for Windows (GraphPad Software, Boston, MA, USA). All analyses were considered statistically significant with *a priori p*-value <0.05.

## 3 Results

### 3.1 Participant characteristics

A total of 14 participants began the investigation. Two participants were unable to complete the off-ice testing protocols and one participant was unable to complete the on-ice testing protocols due to scheduling conflicts. A total of 11 male youth ice hockey players completed the investigation and were included in the statistical analyses. Participant characteristics are presented in Table 1. Of the 11 participants, five were born in the first quartile of the year (Jan–Mar), three were born in each of the second and third quartiles (Apr–June and July–Sep), and no participants were born in the last quartile (Oct–Dec). Descriptive statistics (mean and SD) for all off-ice variables measured are displayed in Table 2, while descriptive statistics for all on-ice tests are presented in Table 3.

**Table 1:** Participants main characteristics (*n* = 11).

Variable	Mean ± SD	Min–Max
Age (years)	10.5 ± 0.6	9.1–11.2
Weight (kg)	35.9 ± 6.3	27.2–45.5
Height (cm)	141.5 ± 6.3	129–152
Wingspan (cm)	139.8 ± 7.4	126–150
Playing hockey experience (years)	4.9 ± 1.1	3.0–6.0
Played hockey in the winter		
Times per week	4.5 ± 0.6	4.0–5.0
Hours per week	5.6 ± 1.2	5.0–7.0
Played hockey in the summer		
Times per week	3.7 ± 1.8	0.0–4.0
Hours per week	4.5 ± 2.2	0.0–6.0

**Table 2:** Off-ice performance variables (*n* = 11).

Variable	Mean ± SD	Min–Max
Muscular strength		
Grip strength – right hand (kg)	21.2 ± 3.8	12–26
Grip strength – left hand (kg)	21.2 ± 3.9	16–28
Grip strength – combined (kg)	42.4 ± 6.9	28–53
Muscular endurance		
Push-ups (reps)	10.1 ± 3.6	2–14
Curl-ups (reps)	19.3 ± 15.6	0–52
Muscular power		
Standing long jump (cm)	161.9 ± 17.9	132–193
Vertical jump (inches)	14.9 ± 2.9	10.5–19.5
Vertical jump (cm)	37.8 ± 7.4	26.7–49.5
One leg squat – right leg (score)	52.7 ± 10.4	40–67
One leg squat – left leg (score)	55.5 ± 14.5	25–75
One leg squat – combined (score)	108.2 ± 23.8	65–138
Agility		
Right – split (s)	3.06 ± 0.17	2.83–3.30
Right – total (s)	5.85 ± 0.31	5.31–6.39
Left – split (s)	2.98 ± 0.19	2.76–3.25
Left – total (s)	5.81 ± 0.30	5.31–6.31
Flexibility		
Trunk flexion (cm)	29.0 ± 7.3	15.0–37.5
Anaerobic fitness		
Mean power (W)	231.7 ± 28.7	192–274
Peak power (W)	320.5 ± 56.5	219–389
Minimum power (W)	176.2 ± 24.9	133–220
Anaerobic capacity (W·kg^−1^)	6.6 ± 0.8	4.9–7.6
Anaerobic power (W·kg^−1^)	8.9 ± 0.8	7.4–10.0
Fatigue index (W·s^−1^)	5.0 ± 1.6	2.2–7.4
Fatigue index (%)	43.8 ± 11.1	18.7–57.1
30 m run		
30 m run – split (6.1 m) sprint time (s)	1.47 ± 0.10	1.30–1.68
30 m run – total (30 m) sprint time (s)	5.46 ± 0.38	4.90–6.13
30 m run – split (6.1 m) sprint speed (m·s^−1^)	4.16 ± 0.29	3.63–4.69
30 m run – total (30 m) sprint average speed (m·s^−1^)	5.52 ± 0.38	4.89–6.12
30 m run – total (30 m) sprint max speed (m·s^−1^)	6.07 ± 0.49	5.29–6.92
30 m run sprint tau (s)	0.49 ± 0.11	0.24–0.63
Aerobic fitness		
Leger stage completed	7.2 ± 1.8	4–10
Estimated VO_2_max (ml·kg^−1^·min^−1^)	54.1 ± 4.4	46.3–59.6

**Table 3:** On-ice performance variables (*n* = 11).

Variable	Mean ± SD	Min–Max
15.2 m – total sprint time (s)	2.00 ± 0.09	1.87–2.15
54 m – split (6.1 m) sprint time (s)	1.53 ± 0.19	1.32–1.89
54 m – total (54 m) sprint time (s)	8.74 ± 0.46	8.01–9.47
15.2 m – total sprint speed (m·s^−1^)	7.60 ± 0.32	7.07–8.13
54 m – split (6.1 m) sprint speed (m·s^−1^)	4.03 ± 0.45	3.23–4.62
54 m – total (54 m) sprint average speed (m·s^−1^)	6.20 ± 0.33	5.70–6.74
54 m – total (54 m) sprint max speed (m·s^−1^)	6.75 ± 0.26	6.23–7.20
54 m sprint tau (s)	0.72 ± 0.25	0.49–1.21
Agility to the right (s)	10.41 ± 0.40	10.01–11.13
Agility to the left (s)	10.67 ± 0.47	9.96–11.54
Shot speed (mph)	38.2 ± 4.0	30.0–44.0
Shot speed (km·h^−1^)	61.4 ± 6.4	48.3–70.8

### 3.2 Correlation between off-ice and on-ice variables

Supplementary Tables S1, S2 show the correlation matrices between off-ice and on-ice variables. The conversion from imperial units to international system units did not affect the correlation coefficients (both yielded identical results, data not shown). Similarly, whether one variable was expressed as the time spent to complete a test (e.g., the seconds to complete the 30 m Run Sprint) or its calculated speed (i.e., m·s^−1^) did not affect the strength, but the direction of the correlation, as expected, neither affected the significance of the results, except for anaerobic capacity which was statistically significantly correlated with on-ice 54 m split (6.1 m) sprint time (*p* = 0.042), but not speed (*p* = 0.052). Of the 33 off-ice variables analyzed, nine were not statistically significantly correlated with any of the on-ice performance variables. Such variables were: height, grip strength (either hand or combined), curl-ups, trunk flexibility, and Wingate-derived absolute power (minimum, mean, and peak). The remaining 24 off-ice variables showed a statistically significant correlation with at least one on-ice performance variable.

The on-ice performance variable that was correlated with most of the off-ice performance variables was the Agility to the Right total time (20 variables), followed by the 15.2 m run time and speed (both 19 variables), Agility to the Left total time (17 variables), 54 m sprint time and average speed (both 15 variables), 54 m sprint max speed (14 variables), 54 m split time and 54 m sprint tau (both 13 variables), 54 m split speed (12 variables), and shot speed (8 variables).

### 3.3 Regression models

The individual regression models from each statistically significantly correlated off-ice performance variable to predict each on-ice performance variable are reported in Supplementary Table S3 through 10. Overall, ten out of the 24 off-ice performance variables that were statistically significantly correlated with on-ice performance variables were included as predictors in at least one model. The following prediction formulas consist of either one or two predictors.

#### 3.3.1 On-ice 15.2 m sprint test

**Figure 3:**
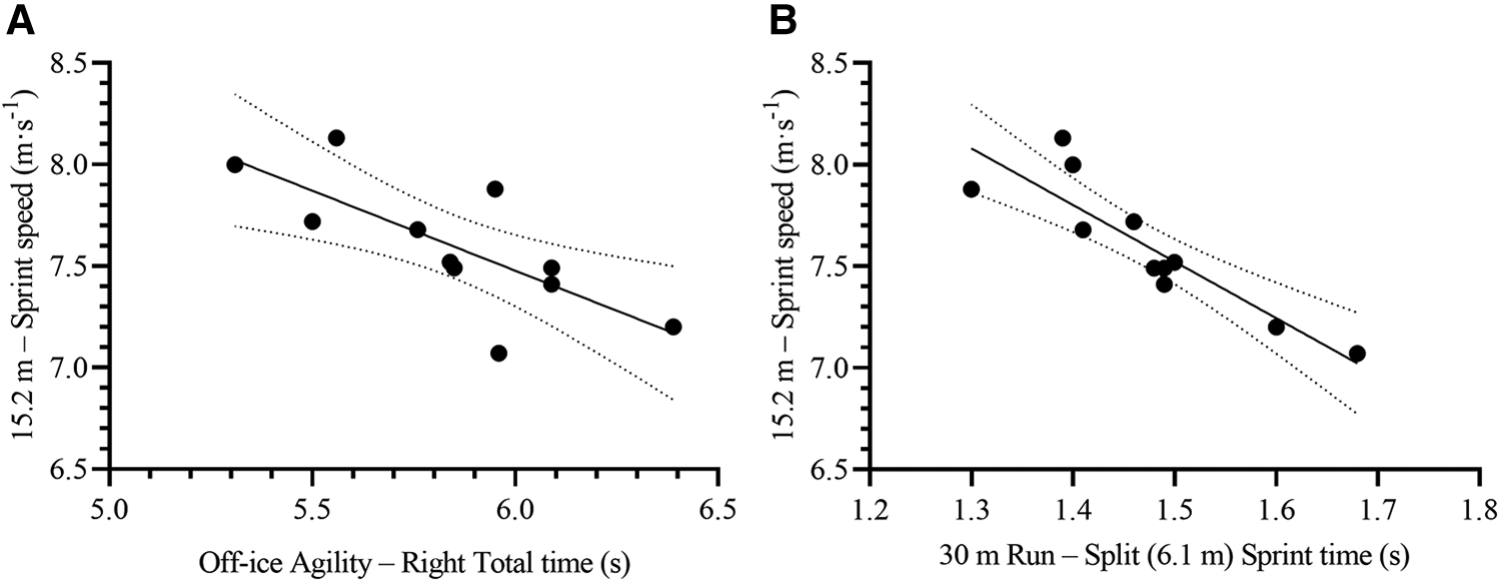
Scatterplots for the correlation between on-ice 15.2 m total sprint speed and its two most relevant off-ice predictors: **(A)** Off-ice agility – right total time, and **(B)** 30 m run – split (6.1 m) sprint time. Each dot represents one participant. The solid lines represent the simple regression line of best fit, while the dotted lines represent the 95% CI for the line of best fit.

The Agility Test to the Right Total time and 30 m Run Split (6.1 m) Sprint time were the most relevant variables to predict on-ice 15.2 m sprint time [F_(2,8)_ = 52.67, *p* < 0.001, R^2^ = 0.93, SEE = 0.03 s, CV = 1.5%, VIF = 1.31] and speed [F_(2,8)_ = 42.87, *p* < 0.001, R^2^ = 0.92, SEE = 0.11 m·s^−1^, CV = 1.4%, VIF = 1.31] (Table 4, Figure 3). The resulting formulas were the following:

$$\begin{array}{r} 15.2\;m\;\mathrm{sprint}\;\mathrm{time}\;(s)\\ \;=0.50+(\mathrm{Agility}\;\mathrm{to}\;\mathrm{the}\;\mathrm{Right}\;\mathrm{Total}\;\mathrm{time}\;[s]\times 0.11)\\ \;+(30\;m\;\mathrm{Run}\;\mathrm{Split}\;\mathrm{Sprint}\;\mathrm{time}\;[s]\times 0.58) \end{array}$$

$$\begin{array}{r} 15.2\;m\;\mathrm{sprint}\;\mathrm{speed}\;(m\cdot s^{-1})\\ \;=13.28-(\mathrm{Agility}\;\mathrm{to}\;\mathrm{the}\;\mathrm{Right}\;\mathrm{Total}\;\mathrm{time}\;[s]\times 0.43)\\ \;-(30\;m\;\mathrm{Run}\;\mathrm{Split}\;\mathrm{Sprint}\;\mathrm{time}\;[s]\times 2.15) \end{array}$$

**Table 4:** Regression coefficients for the most relevant off-ice variables to predict on-ice performance.

Off-ice variable^a^	β	(95% CI)	*p*-value
On-ice 15.2 m sprint time (s)			
Agility right – total (s)	0.11	(0.04, 0.18)	0.006
30 m run – split (6.1 m) sprint time (s)	0.58	(0.38, 0.79)	<0.001
On-ice 15.2 m sprint speed (m·s^−1^)			
Agility right – total (s)	−0.43	(−0.14, −0.72)	0.009
30 m run – split (6.1 m) sprint time (s)	−2.15	(−1.30, −3.00)	<0.001
On-ice 54 m split (6.1 m) sprint time (s)			
30 m run – total (30 m) sprint max speed (m·s^−1^)	−0.340	(−0.214, −0.466)	<0.001
On-ice 54 m split (6.1 m) sprint speed (m·s^−1^)			
30 m run – total (30 m) sprint max speed (m·s^−1^)	0.84	(0.56, 1.13)	<0.001
On-ice 54 m total (54 m) sprint time (s)			
One leg squat – combined (score)	−0.005	(−0.001, −0.010)	0.015
30 m run – total (30 m) sprint max speed (m·s^−1^)	−0.754	(−0.558, −0.951)	<0.001
On-ice 54 m total (54 m) sprint average speed (m·s^−1^)			
One leg squat – combined (score)	0.004	(0.001, 0.007)	0.011
30 m run – total (30 m) sprint max speed (m·s^−1^)	0.527	(0.393, 0.661)	<0.001
On-ice 54 m total (54 m) sprint max speed (m·s^−1^)			
Standing long jump (cm)	0.007	(0.003, 0.011)	0.005
One leg squat – combined (score)	0.007	(0.004, 0.010	<0.001
On-ice 54 m total (54 m) sprint tau (s)			
Anaerobic power (W·kg^−1^)	−0.171	(−0.054, −0.288)	0.010
Leger stage completed	−0.075	(−0.023, −0.127)	0.010
On-ice agility to the right time (s)			
30 m run – split (6.1 m) sprint time (s)	1.56	(0.12, 2.99)	0.037
30 m run – total (30 m) sprint time (s)	0.64	(0.25, 1.02)	0.005
On-ice agility to the left time (s)			
Agility left – split (s)	1.08	(0.28, 1.88)	0.014
30 m run – split (6.1 m) sprint time (s)	2.92	(1.48, 4.37)	0.002
On-ice shot speed (km·h^−1^)			
30 m run – split (6.1 m) sprint speed (m·s^−1^)	−17.6	(−7.3, −27.9)	0.004

^a^ The off-ice variables reported were selected as the most relevant to predict the on-ice variable immediately above them using the forward method in linear regression.

#### 3.3.2 On-ice 54 m split (6.1 m) sprint test

**Figure 4:**
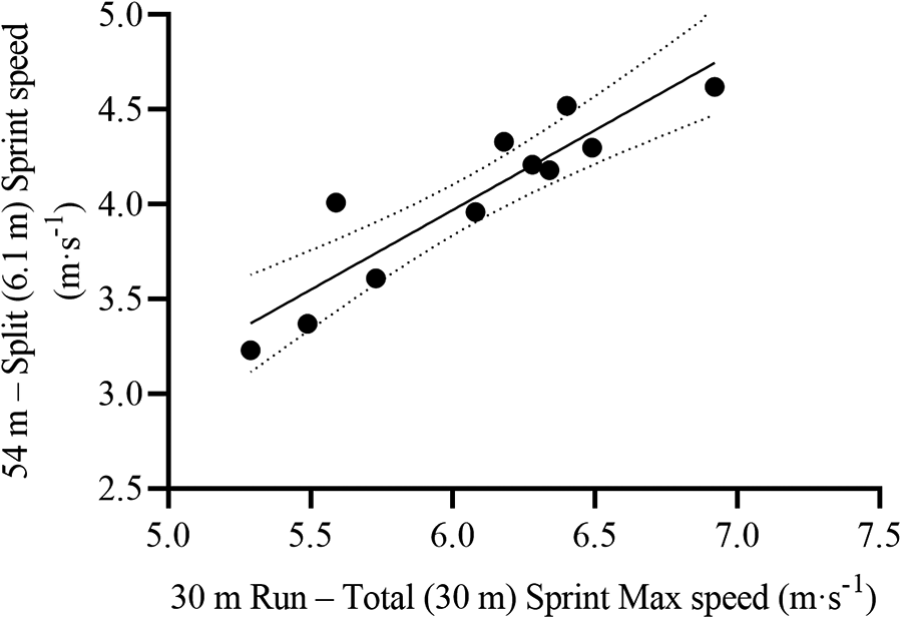
Scatterplot for the correlation between on-ice 54 m split (6.1 m) sprint speed and its most relevant off-ice predictor, 30 m Run total sprint Max speed. Each dot represents one participant. The solid line represents the simple regression line of best fit, while the dotted lines represent the 95% CI for the line of best fit.

The 30 m Run Total Sprint Max speed was the single and most relevant variable to predict on-ice 54 m split (6.1 m) sprint time [F_(1,9)_ = 37.23, *p* < 0.001, R^2^ = 0.81, SEE = 0.09 s, CV = 5.9%] and speed [F_(1,9)_ = 45.11, *p* < 0.001, R^2^ = 0.83, SEE = 0.20 m·s^−1^, CV = 5.0%] (Table 4, Figure 4). The resulting formulas were the following:

$$\begin{array}{r} 54\;m\;\mathrm{split}\;\mathrm{sprint}\;\mathrm{time}\;(s)\\ \;=3.599-(30\;m\;\mathrm{Run}\;\mathrm{Total}\;\mathrm{Sprint}\;\mathrm{Max}\;\mathrm{speed}\;[m\cdot s^{-1}]\times 0.34) \end{array}$$

$$\begin{array}{r} 54\;m\;\mathrm{split}\;\mathrm{sprint}\;\mathrm{speed}\;(m\cdot s^{-1})\\ \;=-1.09+(30\;m\;\mathrm{Run}\;\mathrm{Total}\;\mathrm{Sprint}\;\mathrm{Max}\;\mathrm{speed}\;[m\cdot s^{-1}]\times 0.84) \end{array}$$

#### 3.3.3 On-ice 54 m total sprint test

**Figure 5:**
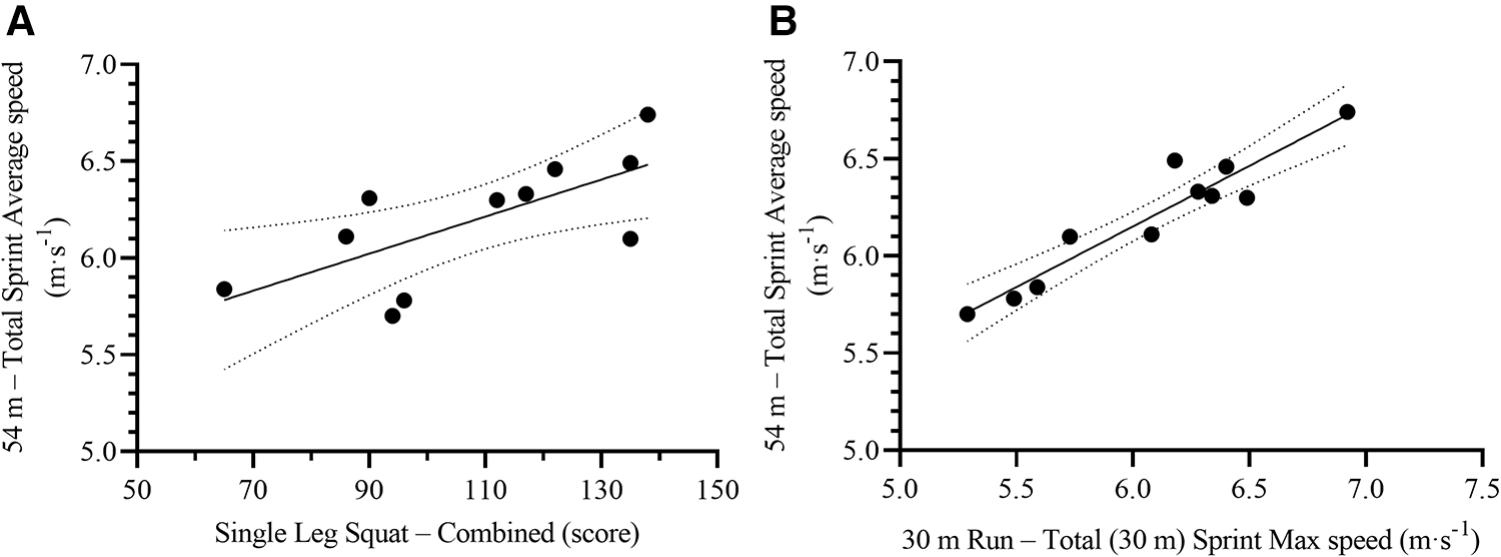
Scatterplots for the correlation between on-ice 54 m total sprint average speed and its most relevant off-ice predictors: **(A)** One leg squat – combined, and **(B)** 30 m run – total sprint max speed. Each dot represents one participant. The solid lines represent the simple regression line of best fit, while the dotted lines represent the 95% CI for the line of best fit.

**Figure 6:**
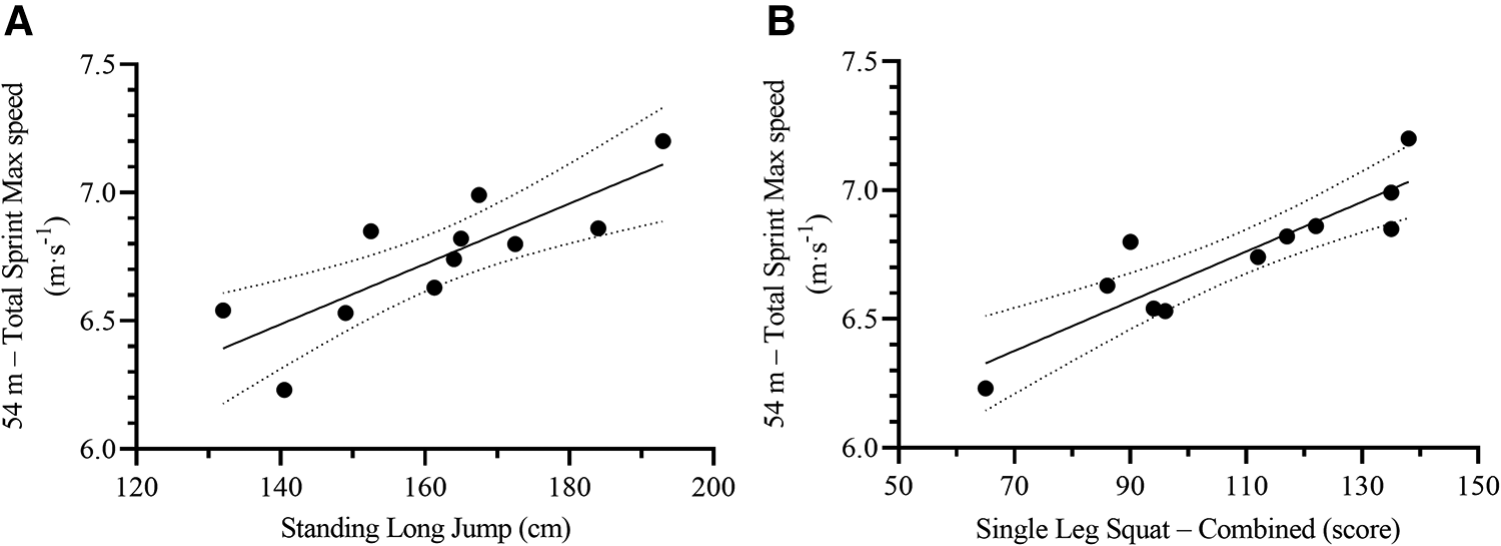
Scatterplots for the correlation between on-ice 54 m total sprint max speed and its most relevant off-ice predictors: **(A)** standing long jump, and **(B)** One Leg squat – combined. Each dot represents one participant. The solid lines represent the simple regression line of best fit, while the dotted lines represent the 95% CI for the line of best fit.

The One Leg Squat Combined and 30 m Run Total Sprint Max speed were the most relevant variables to predict on-ice 54 m total (54 m) sprint time [F_(2,8)_ = 79.70, *p* < 0.001, R^2^ = 0.95, SEE = 0.11 s, CV = 1.3%, VIF = 1.37], and average speed [F_(2,8)_ = 84.74, *p* < 0.001, R^2^ = 0.96, SEE = 0.08 m·s^−1^, CV = 1.3%, VIF = 1.37] (Table 4, Figure 5); whereas Standing Long Jump and One Leg Squat Combined were the most relevant to predict max speed [F_(2,8)_ = 49.03, *p* < 0.001, R^2^ = 0.93, SEE = 0.08 m·s^−1^, CV = 1.2%, VIF = 1.52] (Table 4, Figure 6). The resulting formulas were the following:

$$\begin{array}{r} 54\;m\;\mathrm{total}\;\mathrm{sprint}\;\mathrm{time}\;(s)\\ \;=13.906-(\mathrm{One}\;\mathrm{Leg}\;\mathrm{Squat}\;\mathrm{Combined}\;[\mathrm{score}]\times 0.005)\\ \;-(30\;m\;\mathrm{Run}\;\mathrm{Total}\;\mathrm{Sprint}\;\mathrm{Max}\;\mathrm{speed}\;[m\cdot s^{-1}]\times 0.754) \end{array}$$

$$\begin{array}{r} 54\;m\;\mathrm{total}\;\mathrm{sprint}\;\mathrm{average}\;\mathrm{speed}\;(m\cdot s^{-1})\\ \;=2.570+(\mathrm{One}\;\mathrm{Leg}\;\mathrm{Squat}\;\mathrm{Combied}\;[\mathrm{score}]\times 0.004)\\ \;+(30\;m\;\mathrm{Run}\;\mathrm{Total}\;\mathrm{Sprint}\;\mathrm{Max}\;\mathrm{speed}\;[m\cdot s^{-1}]\times 0.527) \end{array}$$

$$\begin{array}{r} 54\;m\;\mathrm{total}\;\mathrm{sprint}\;\max\;\mathrm{speed}\;(m\cdot s^{-1})\\ \;=4.945+(\mathrm{Standing}\;\;\mathrm{Long}\;\mathrm{Jump}\;[\mathrm{cm}]\times 0.007)\\ \;+(\mathrm{One}\;\mathrm{Leg}\;\mathrm{Squat}\;\mathrm{Combied}\;[\mathrm{score}]\times 0.007) \end{array}$$

#### 3.3.4 On-ice 54 m sprint tau

**Figure 7:**
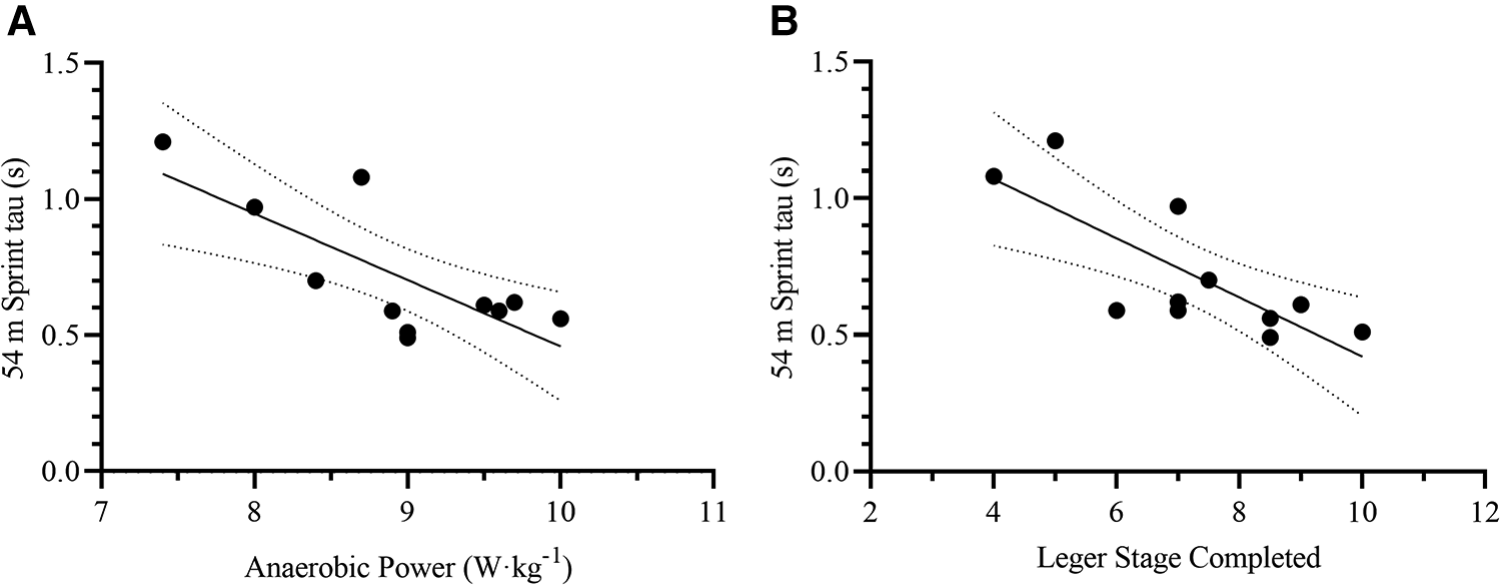
Scatterplots for the correlation between on-ice 54 m total sprint tau and its two most relevant off-ice predictors: **(A)** anaerobic power, and **(B)** leger stage completed. Each dot represents one participant. The solid lines represent the simple regression line of best fit, while the dotted lines represent the 95% CI for the line of best fit.

Initially, the forward method included the Anaerobic Power, Leger Stage Completed, and Estimated VO_2_max as the most relevant variables to predict on-ice 54 m sprint tau. Even though the model showed a high R^2^ (0.91) and low SEE (0.09), the collinearity of VO_2_max with the Leger Stage was large (VIF = 19.98). Therefore, we excluded the Estimated VO_2_Max from the variable list and re-ran the analysis, leading to the inclusion of Anaerobic Power and Leger Stage Completed as the most relevant variables to predict on-ice 54 m sprint tau [F_(2,8)_ = 19.66, *p* < 0.001, R^2^ = 0.83, SEE = 0.11 s, CV = 15.3%, VIF = 1.22] (Table 4, Figure 7). The resulting formula was the following:

$$\begin{array}{r} 54\;m\;\mathrm{total}\;\mathrm{sprint}\;\mathrm{tau}\;(s)\\ \;=2.790-(\mathrm{Anaerobic}\;\mathrm{Power}\;[W\cdot kg^{-1}]\times 0.171)\\ \;-(\mathrm{Leger}\;\mathrm{Stage}\;\mathrm{Completed}\times 0.075) \end{array}$$

#### 3.3.5 On-ice agility to the right test

**Figure 8:**
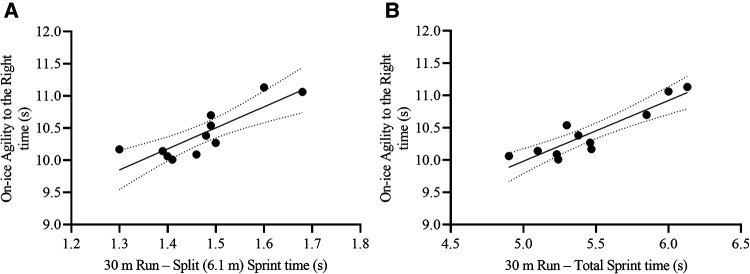
Scatterplots for the correlation between on-ice agility to the right total time and its two most relevant off-ice predictors: **(A)** 30 m run – split (6.1 m) sprint time, and **(B)** 30 m run – total sprint time. Each dot represents one participant. The solid lines represent the simple regression line of best fit, while the dotted lines represent the 95% CI for the line of best fit.

Initially, the forward method included the 30 m Run Total Sprint time and 30 m Run Total Max speed as the most relevant variables to predict on-ice Agility to the Right time. Even though the model showed a high R^2^ (0.92) and low SEE (0.12), the collinearity of Total Sprint Max speed with the Total Sprint time was large (VIF = 12.65). Therefore, we excluded the 30 m Run Total Max speed from the variable list and re ran the analysis, leading to the inclusion of 30 m Run Total Sprint time and 30 m Run Split Sprint time as the most relevant variables to predict on-ice Agility to the Right time [F_(2,8)_ = 36.10, *p* < 0.001, R^2^ = 0.90, SEE = 0.14 s, CV = 1.3%, VIF = 2.09] (Table 4, Figure 8). The resulting formula was the following:

$$\begin{array}{r} \mathrm{Agility}\;\mathrm{to}\;\mathrm{the}\;\mathrm{Right}\;\mathrm{Total}\;\mathrm{time}\;(s)\\ \;=4.65+(30\;m\;\mathrm{Run}\;\mathrm{Split}\;\mathrm{Sprint}\;\mathrm{time}\;[s]\times 1.56)\\ \;+(30\;m\;\mathrm{Run}\;\mathrm{Total}\;\mathrm{Sprint}\;\mathrm{time}\;[s]\times 0.64) \end{array}$$

#### 3.3.6 On-ice agility to the left test

**Figure 9:**
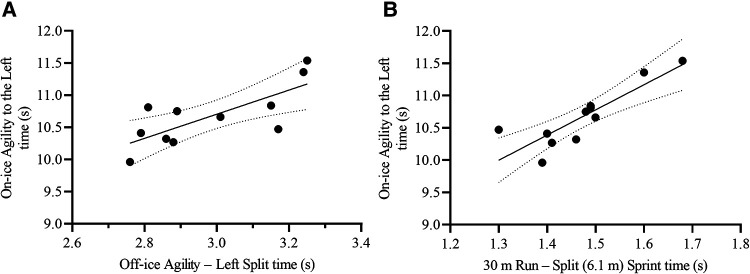
Scatterplots for the correlation between on-ice agility to the left total time and its two most relevant off-ice predictors: **(A)** Off-ice agility – left split time, and **(B)** 30 m run – split (6.1 m) sprint time. Each dot represents one participant. The solid lines represent the simple regression line of best fit, while the dotted lines represent the 95% CI for the line of best fit.

The off-ice Agility to the Left split time and 30 m Run Split (6.1 m) Sprint time were the most relevant variables to predict on-ice Agility to the Left time [F_(2,8)_ = 30.44, *p* < 0.001, R^2^ = 0.88, SEE = 0.18 s, CV = 1.7%, VIF = 1.33] (Table 4, Figure 9). The resulting formula was the following:

$$\begin{array}{r} \mathrm{Agillity}\;\mathrm{to}\;\mathrm{the}\;\mathrm{Left}\;\mathrm{Total}\;\mathrm{time}\;(s)\\ \;=3.15+(\mathrm{Agility}\;\mathrm{to}\;\mathrm{the}\;\mathrm{Left}\;\mathrm{Split}\;\mathrm{time}\;[s]\times 1.08)\\ \;+(30\;m\;\mathrm{Run}\;\mathrm{Split}\;\mathrm{Sprint}\;\mathrm{time}\;[s]\times 2.92 \end{array})$$

#### 3.3.7 On-ice shot speed test

**Figure 10:**
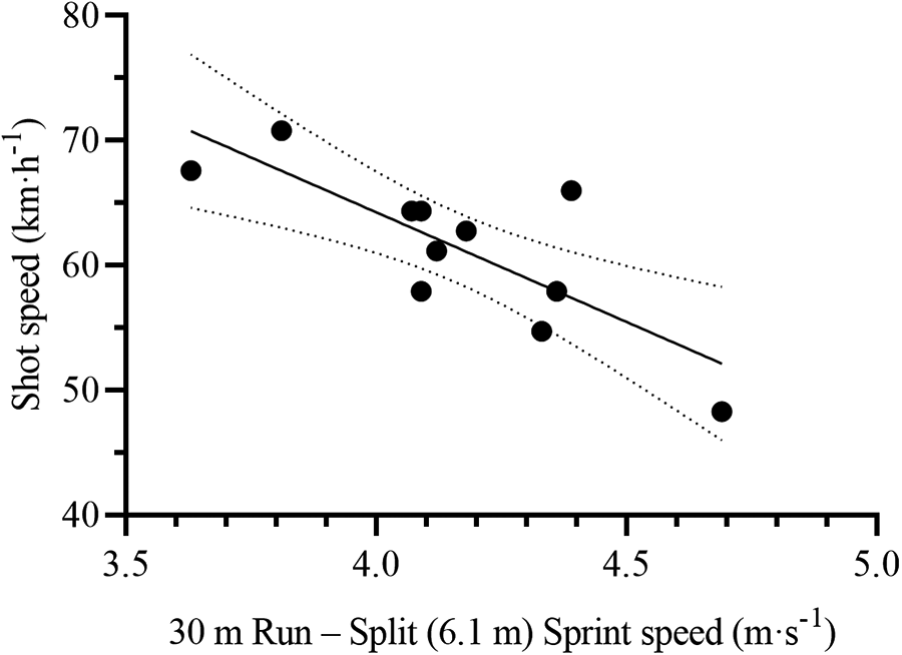
Scatterplot for the correlation between on-ice shot speed and its most relevant off-ice predictor: 30 m Run – split (6.1 m) sprint speed. Each dot represents one participant. The solid line represents the simple regression line of best fit, while the dotted lines represent the 95% CI for the line of best fit.

The 30 m Run Split (6.1 m) Sprint speed was the single and most relevant variable to predict on-ice shot speed [F_(1,9)_ = 15.03, *p* = 0.004, R^2^ = 0.63, SEE = 4.1 km·h^−1^, CV = 6.7%] (Table 4, Figure 10). The resulting formula was the following:

$$\begin{array}{r} \mathrm{Shot}\;\mathrm{Speed}\;(\mathrm{km}\cdot h^{-1})\\ \;=134.7-(30\;m\;\mathrm{Run}\;\mathrm{Split}\;\mathrm{Sprint}\;\mathrm{speed}\;[m\cdot s^{-1}]\times 17.6) \end{array}$$

## 4 Discussion

The primary purpose of this study was to examine the relationship between off-ice physical fitness performance and on-ice sport-related performance in male youth ice hockey players. Both off-ice and on-ice testing measures may be used to assess qualities that relate to game performance; however, the research to date is particularly limited in youth (21, 22). To our knowledge, this is the first study to examine a comprehensive battery of measures in male ice hockey players under the age of 12 years. We demonstrated that several off-ice measures can predict on-ice performance in the youth male ice hockey players. In fact, the majority of the off-ice variables (72.7%) were significantly associated with at least one of the 11 on-ice performance measures. Furthermore, our analysis identified 10 key off-ice determinants of on-ice performance including sprinting speed and acceleration, agility, anaerobic power, standing long jump, aerobic fitness, and one leg squat. During this study, we also revealed important relationships between maximal sprinting velocity and acceleration in both on-ice and off-ice settings in youth male ice hockey players.

### 4.1 Predictors of performance

Various research studies have looked at the best indicators correlated to skating speed and/or hockey performance as being sprint running times (23–31), vertical jump (24, 25, 28, 32–35), horizontal jump (4, 25, 34, 36–38), anaerobic Wingate (4, 21, 25, 34, 36, 39), body composition (21, 32, 36, 39, 40), leg strength/endurance (21, 29, 30, 41, 42), upper body strength/endurance (21, 30, 36, 42), and balance (26). However, limited research has been conducted with youth ice hockey players with the majority of work in young cohorts involving players older than 13 years [e.g., from the 14U, 16U, 18U, and Junior levels (2, 13, 22)].

In the current investigation, we were able to examine the key off-ice predictors of on-ice performance (as evaluated by skating speed, skating acceleration, skating agility, and shot velocity). The off-ice tests were chosen for inclusion based on the premise that they are used in the combine measures for NHL entry draft players (7) and/or are often used in high performance hockey settings (e.g., the NHL). These off-ice tests included anthropometrics, musculoskeletal fitness (muscular strength, muscular endurance, muscular power, and flexibility), anaerobic power, and aerobic fitness. Of the 33 off-ice variables assessed, 24 demonstrated a statistically significant correlation with at least one on-ice performance variable. Nine off-ice variables [i.e., height, grip strength (either hand or combined), curl-ups, trunk flexibility, and Wingate-derived absolute power (minimum, mean, and peak)] were not significantly correlated with any of the on-ice performance variables. The on-ice performance measure that demonstrated the greatest number of significant correlations with off-ice performance variables was Agility to the Right (20 variables). Additionally, notable correlations were observed with several off-ice performance measures, with the 15.2 m run time and speed being most prominent, involving 19 variables each. This was followed by Agility to the Left total time (17 variables), 54 m sprint time, and average speed (each involving 15 variables), maximum speed during the 54 m sprint (14 variables), 54 m split time, and 54 m sprint relative acceleration (tau) (each involving 13 variables), 54 m split speed (12 variables), and shot speed (8 variables).

### 4.2 Speed, acceleration, and agility

It is widely accepted that mastering acceleration, speed, and agility on the ice is crucial for hockey players, regardless of sex or age (43, 44). The fast-paced nature of ice hockey requires athletes to stop, start, accelerate, and change direction quickly and forcefully (45). Skating acceleration, speed, and agility are thought to provide important in-game performance advantages (such as quickly transitioning from defense to offense, chasing down opponents, and creating more scoring opportunities) particularly at the elite level (2, 21, 29, 46, 47). Previous studies have demonstrated the importance of skating acceleration, speed, and/or agility in ice hockey in largely older male and female samples (23–30, 41, 48, 49). Research has also revealed that sprinting skating performance (e.g., sprinting speed and acceleration) is enhanced in high caliber vs. lower caliber ice hockey players (44, 50–53). For instance, Renaud and colleagues (51) revealed that higher caliber skaters were able to achieve greater vertical centre of mass acceleration during each stride that allowed for greater horizontal traction, forward propulsion, lower double-support times, and faster starts with higher stride rates. Douglas and colleagues (54) recently revealed that the ability to accelerate and skate at higher speeds was a significant discriminator between elite and sub-elite female ice hockey players.

Off-ice sprinting has been argued to share biomechanical similarities to on-ice skating performance (55). The ability to accelerate and sprint on land have consistently been shown to predict skating performance in high school, collegiate, and elite junior male and female ice hockey players (24–26, 41, 48, 56). For example, Janot and colleagues (24) revealed that running speed was a significant predictor of skating performance in Division III collegiate female and male (age = 20.5 ± 1.4 years) ice hockey players. Recently, Thompson and coworkers (56) reported that off-ice resisted sprints were better predictors of on-ice skate performance compared to commonly used off-ice fitness tests. The authors argued that resisted sprint tests showed strong associations with on-ice sprints, making them valuable indicators of acceleration ability during periods of limited access to on-ice facilities. Pal'ov et al. (57) revealed that off-ice 40 m running was a strong predictor of 40 m on-ice skating performance in Junior ice hockey players.

Limited research has been conducted in young populations (35). Bracko and George (31) showed that the 40-yd dash (36.6 m) was a strong predictor of skating speed (up to 44 m) and on-ice repeat sprint ability in female ice hockey players (aged 8–16 years). In our current study, we revealed that off-ice measures of maximal speed, acceleration, and agility were strong predictors of on-ice measures of agility, speed, and shot velocity (see Supplementary Tables). Therefore, our current findings build upon previous literature demonstrating the importance of off-ice speed, acceleration, and agility in predicting on-ice performance.

Authors have increasingly argued for the evaluation of on-ice skating kinetics (such as force, velocity, and power) for talent identification and personalized training programs (58). Several authors have examined the sprint mechanical force-velocity properties of ice hockey players (58–64). This includes research demonstrating the reliability of force-velocity-power variables during ice hockey sprint acceleration (58, 63). Team sports athletes are thought to achieve maximal speed earlier during a sprint (e.g., 30–40 m) vs. track and field sprinters (e.g., 50–60 m) (19).

Unique to our study, we were able to capture on-ice and off-ice sprinting split times to determine sprint mechanical force-velocity properties of young male ice hockey players [including modelling using the mono-exponential equation involving maximum sprinting speed and relative acceleration (tau)] (19). We found important relationships between maximal sprinting velocity and acceleration in both on-ice and off-ice settings in youth male ice hockey players. This work builds upon other research with high performance athletes in older highly trained female (58–62) and male (63) ice hockey players and athletes from other sports (65, 66). It should be highlighted however that researchers (67) have recently argued against the utility of force-velocity profiling during sprinting activities.

### 4.3 Musculoskeletal fitness

Our current study examined multiple off-ice musculoskeletal fitness measures including muscular power (standing long jump and vertical jump), muscular strength (grip strength and one leg squat), muscular endurance (push-ups and curl-ups), and flexibility (trunk forward flexion) in male youth ice hockey players. Our findings revealed that most of our musculoskeletal variables were predictive of on-ice performance. The exceptions were grip strength (either hand or combined), curl-ups, and trunk flexibility. Overall, these findings build upon previous research demonstrating the importance of musculoskeletal fitness for optimal hockey performance in young and adult ice hockey players (4, 21, 24, 25, 28–30, 32–38, 41, 42, 68).

### 4.4 Muscular power

Vertical jump has been shown by several authors to predict on-ice hockey performance (24, 25, 28, 32–35, 68). Athletes with more explosive leg power during the vertical jump are often thought to possess an increased capacity for on-ice acceleration and maximal sprinting velocity. For instance, Janot and colleagues (24) revealed that the vertical jump was a significant predictor of skating performance in National Collegiate Athletic Association (NCAA) Division III collegiate female and male ice hockey players. Mascaro et al. (28) revealed that the vertical jump was the single best predictor of skating speed in professional ice hockey players. Gupta et al. (68) recently examined the relationship between three different vertical jump tests (countermovement jump with arm swing, squat jump, and depth drop jump) and ice-skating performance (assessed via by the skating multistage aerobic test, forward and backward acceleration test, top speed test, and repeated sprint ability test) in junior male ice hockey players. The authors reported that all vertical jump measures (in particular depth drop jump) were significantly correlated with on-ice skating performance measures (with the exception) of the repeated sprint ability test. However, others have demonstrated limited associations between vertical jump and sport performance. For instance, Kniffin et al. (32) examined the relationships between off-ice strength and conditioning measures with playing performance indicators in NCAA Division 1 Men's Ice Hockey players over a 14-year period. The authors revealed that vertical jump did not show a significant relationship with points scored, but vertical jump did show a significant positive relationship with games played.

In our current study, vertical jump was predictive of several off-ice performance measures including skating split and total speed, sprinting tau, and agility. These findings support previous research demonstrating that the vertical jump is associated with skating speed and agility. This finding is thought to be related to the common muscle groups and angles of motion engaged during the skating stride and the vertical jump. Contreras and colleagues (69) argued that the muscles of the posterior chain (particularly the hip extensors) are very important in the generation of maximum speed and power during sprinting and jumping activities. According to Kaartinen and colleagues (70) the vastus lateralis, gluteus maximus, and soleus are highly active during the propulsive phase of skating, and the biceps femoris is highly active during the glide phase of skating; therefore, similar to the vertical jump, a large component of the skating performance is created from the hip and ankle.

Horizontal jump testing has increasingly been recommended in the assessment of speed athletes (including ice hockey players) (25). The standing long jump is a skillful test that measures the explosive power of the legs, in addition to coordination of the whole body when taking off, during flight, and when landing. When jumping, there is a multi-dimensional component involved, where the participants explosively push themselves in both an upward and forward direction. This is much different than the vertical jump where the participant travels in only one plane. Contreras (69) proposed that horizontal movements elicit more activation from the gluteal and hamstrings than vertical movements, which may lead to greater transfer to horizontal power. Nagano et al. (71) found that horizontal jumping also used more gluteal and hamstring activation as compared to vertical jumping. Moreover, Kotsifaki and coworkers (72) examined the horizontal hop test and concluded that with the horizontal hop, the relative work contribution from the hip, knee, and ankle joints were 44.3%, 12.9%, and 42.8%, respectively. Therefore, the lower body muscle activation patterns for the standing long jump and skating have marked similarities.

Various researchers have demonstrated the relationship between horizontal jump power and skating and/or hockey performance (4, 25, 34, 36–38). Our current findings revealed that the standing long jump was a significant and strong predictor of on-ice skating ability including sprinting, acceleration, and agility. The strength of the relationship supports the arguments of Farlinger and colleagues (25) who stated that coaches should include measures of horizontal power (such as off-ice sprint and three hop jump) to assess skating capability. In their work, they demonstrated that the standing long jump was moderately correlated to sprint performance and cornering capability in Bantam, Midget, and Major Midget players (25). Henriksson and colleagues (73) reported how the single leg standing long jump explained a significant amount of the variance in skating performance.

### 4.5 Muscular endurance

Measures of muscular endurance (such as push-ups, chin-ups, and curl-ups) have frequently been included within high performance (e.g., NHL and Junior) and developmental assessment protocols for ice hockey (7, 74). Also, muscular endurance measures (i.e., push-ups) have been shown to be predictive of time to injury in male and female varsity athletes competing in basketball, volleyball, and ice hockey (75).

Previous research has examined the relationship between measures of muscular endurance and on-ice performance. This research, largely in adult populations, has demonstrated the important role of muscular endurance for on-ice performance (25, 42). Example measures of muscular endurance that have been associated with on-ice performance include push-ups (25), curl-ups (42), leg press (42), chin-ups (42), and bench press (42). It is important to highlight that when multiple measures of muscular endurance are taken one measure often provides a better prediction of off-ice performance than the other(s) (42). For instance, Peyer and colleagues (42) revealed that game plus/minus was significantly correlated with sprint repeat (*r* = 0.57), leg press (*r* = 0.55), bench press (*r* = 0.50), and chin-ups (*r* = 0.46). Moreover, there are reports of limited relationships between off-ice measures of muscular endurance and on-ice performance (31).

In the present investigation, push-ups were significantly associated with on-ice acceleration (including relative acceleration), speed, and agility. This is similar to the findings of Bracko and Fellingham (35) who revealed that in youth male ice hockey players between the ages of 10–14 years (mean age = 12.5 years) vertical jump measures and push-ups were reasonable predictors of skating acceleration, speed, and full speed.

Our current study revealed no significant relationships between curl-ups and any on-ice performance measure. This is supported by other studies demonstrating low to moderate relationships in younger (31) and older ice hockey players (76). However, core stability is believed to be important for reducing the risk for musculoskeletal injury in ice hockey players (6).

### 4.6 Muscular strength

Previous studies have demonstrated the importance of leg strength (21, 29, 30, 41, 42, 44) and upper body strength (21, 30, 36, 42) for on-ice hockey performance. It should be highlighted that many investigations took measures that would be classically defined as assessments of muscular endurance (e.g., tests involving repetitions to fatigue or repetitions within a certain time frame).

Grip strength has been associated with team success at the elite levels (such as the NHL) (6) although there is discrepancy in the area [perhaps owing to the more homogeneous nature of elite NHL athletes (4)]. Hoff and colleagues (30) reported that strength and body mass were the main differences seen between elite and junior ice hockey players. Peterson and coworkers (77) also revealed that NCAA Division I players had higher grip strength than Division III counterparts. Tarter and colleagues revealed that approximately 35% of the variance in NHL draft selection could be explained via an aggregate upper body strength score (11); whereas, Vescovi et al. (78) revealed that off-ice fitness testing performance did not predict NHL draft selection.

Limited data exists at the youth level (38, 79, 80). Lemoyne and colleagues (79) revealed that a series of musculoskeletal measures (including broad jump, vertical jump, medicine ball throw, grip strength, and chin-ups) did not discriminate the male athletes selected or not selected to Team Quebec (U16). However, selected female players displayed higher fitness (VO_2_max, 30 m running sprint, off-ice agility, broad jump, vertical jump, and chin-ups), on-ice sprinting, acceleration, and agility, and psychological characteristics. Our mean grip strength data compares well to recent normative data developed for male and female youth hockey players (81). In our study, grip strength was not significantly correlated with any of the on-ice performance variables. However, this should not negate the importance of muscular strength for on-ice performance, particularly considering the other indictors of muscular strength and endurance that were associated with on-ice performance in this study. Based on our findings, it may be advisable to include other measures of muscular strength when working with young athletes. It is plausible that grip strength may not be as predictive of on ice performance at younger ages compared to older ages owing to the changes in handgrip strength that comes with aging and maturation (82). Measures of peak leg strength may provide additional insight when evaluating young ice hockey players. For instance, Budarick and colleagues (44) revealed that peak leg strength was a strong predictor of peak skating speed.

The single leg squat is a test that evaluates unilateral leg strength, balance, and full body coordination (7). The single leg squat has been used extensively for years in the training of high-performance athletes, including professional hockey players. This is largely owing to the belief that this exercise will improve strength, power, stability, and coordination, help reduce muscle asymmetries, and potentially support injury prevention within ice hockey. To our knowledge, no study has evaluated the single leg squat test in combination with other off-ice and on-ice performance measures in ice hockey players, despite its previous inclusion in the NHL draft combine (7).

Our findings revealed that single leg squat (both sides individually and combined) was significantly correlated with several on-ice performance measures including on-ice sprinting speed and agility. As such, enhanced single leg squat was associated with improved on-ice sport performance. However, there was no relationship with acceleration measures [including relative acceleration (tau)] during the 54 m on-ice sprint test. Further research is warranted regarding the utility of the single leg squat test in ice hockey settings. It is important to highlight that investigators have increasingly included single leg measures of muscular power in assessment protocols demonstrating a strong relationship with on-ice skating performance (73).

### 4.7 Flexibility

Limited research has examined flexibility and its relationship to on-ice performance indicators in ice hockey, particularly youth ice hockey players. Previous work has demonstrated that flexibility may vary according to position being greater in goalies than forwards and defensemen (6). Also, others postulate that greater flexibility may help prevent and/or reduce injury (83). Our current findings demonstrate that flexibility (evaluated by the forward flexion test) had little relationship to on-ice performance. These findings are consistent with other studies demonstrating limited predictive capability of flexibility for on-ice performance in adult ice hockey players (26, 31, 78). Based on our current findings and that of others, it could be inferred that flexibility, while an important aspect of physical fitness to help prevent and reduce injury (83), is not a critical factor in on-ice performance in youth ice hockey players that play forward or defense.

### 4.8 Anaerobic Wingate test

Various studies have revealed the relationship between Wingate derived measures of anaerobic power and fitness and on-ice performance markers (4, 21, 25, 34, 36, 39). For instance, previous research with older athletes has shown that on-ice sprint time is related to anaerobic power as evaluated by the Wingate anaerobic test (25, 34, 39, 84, 85). Also, peak anaerobic power (evaluated during a 30-s Wingate test) has been shown to be an important predictor of NHL draft entry position (4). Roczniok and colleagues (86) revealed that measures of anaerobic power and capacity were predictive of players selected by expert coaches to a team in the top division of a Polish ice hockey league. However, questions have been raised about the utility of anaerobic Wingate testing for predicting game performance in ice hockey. For instance, Peterson and colleagues (77) demonstrated that NCAA Division 1 hockey players were able to generate more power than Division III players during off-ice performance tests (including vertical jump and Wingate peak power). In another paper (85), these authors revealed that off-ice anaerobic power tests could predict on-ice acceleration and top speed, but not on-ice repeated shift performance. The authors argued that off-ice anaerobic power tests may not be good predictors of the repeated shift ability of the player or performance in ice hockey.

The majority of this research has been conducted in adult populations with limited evidence from younger ice hockey players. For instance, Farlinger et al. (25) noted that the Wingate results of mean watt output and peak watt output were significantly correlated to on-ice sprint speed in elite Bantam, Midget, and Major Midget athletes. In our study, Wingate-derived absolute power measures (minimum, mean, and peak) were not significantly correlated with any of the on-ice performance variables. However, Wingate anaerobic power and capacity in relative terms were significant predictors of on-ice skating speed, acceleration, and/or agility. In our study, the two most relevant off-ice predictors for on-ice 54 m total sprint relative acceleration (tau) were anaerobic power and aerobic fitness. Accordingly, our results support that of others demonstrating the important predictive value for Wingate-derived anaerobic fitness for on-ice speed, acceleration, and agility in male youth ice hockey players. For instance, our findings compared well to the recent work of Glaude-Roy and colleagues (64) who examined the relationships between the sprinting force-velocity and anaerobic capacities (via Wingate anaerobic power and repeat sprint tests) in adolescent male and female ice hockey players. The authors reported that the ability to apply a force and low and high intensities are related to anaerobic performance on a cycle ergometer and on-ice.

### 4.9 Aerobic fitness

The relationship between aerobic fitness and on-ice hockey performance has been evaluated extensively in later minor hockey age groups and competitive junior and professional populations. Recent studies have established that the exercise intensities of ice hockey reflect both significant aerobic and anaerobic energy contributions during game play (87, 88). The assessment of aerobic fitness (often via an incremental VO_2_max test) is included in arguably the majority of testing combines (including the NHL entry draft combine) (7). It has been postulated that a high aerobic fitness is of benefit for game performance for oxygen delivery and energy production (via oxidative phosphorylation), by delaying the onset of fatigue, and/or enhancing recovery during games (87–89). It is widely accepted that developing both anaerobic and aerobic fitness provides performance advantages in ice hockey.

There is conflicting evidence regarding the predictive value of aerobic fitness for skating and in-game performance. For instance, some researchers have not observed a significant relationship between off-ice VO_2_max and on-ice VO_2_max and/or performance (22). Durocher and colleagues (90) revealed no significant relationship between VO_2_max values determined off-ice (cycle ergometry) vs. on-ice (graded on-ice skating) arguing against the usage of off-ice VO_2_max testing in the NHL entry draft combine. Carey and coworkers (91) also reported no significant correlation between off-ice VO_2_max (treadmill via the modified Bruce protocol) and on-ice recovery from a repeat sprint test. In comparison, other studies with female and male ice hockey players have demonstrated the important relationship between aerobic fitness and on-ice performance measures (88). Importantly, the relationship between aerobic fitness and skating performance may vary based on sex (41).

The findings from our current investigation build upon the current literature. The VO_2_max of the athletes (54.1 ± 4.4 ml·kg^−1^·min^−1^) from our study compare well to other studies with older minor hockey male athletes (22) and adult male ice hockey players (88). Our current study revealed that aerobic fitness was a strong predictor of maximal skating speed, acceleration, and agility. Those athletes with the highest aerobic fitness had the fastest maximal speed, acceleration, and agility. Aerobic fitness explained from 38% to 77% of the variance in on-ice skating performance. We also revealed that aerobic fitness (along with anaerobic power) was one of the two most relevant off-ice predictors for relative acceleration (tau) during the 54 m on-ice sprint. These findings support those of others demonstrating the important relationships between off-ice aerobic fitness and on-ice skating performance (24, 41, 73, 88), team selection (86), and in-game performance (89) in older ice hockey players. For instance, Roczniok and et al. (86) revealed that VO_2_max was amongst the best predictors of success in the selection of top level adult ice hockey players. In a longitudinal study (3 years), Green and colleagues (89) revealed a moderate correlation (explaining 17% of the variance) between VO_2_max and net scoring chances in NCAA Division I hockey players. However, our findings are contrary to the findings of Allisse and colleagues (22) who demonstrated no-significant relationships between VO_2_max and skating performance in older minor hockey players.

### 4.10 Anthropometrics

Several research studies have demonstrated the importance of body composition (such as body mass, height, lean body mass, and/or body fat percentage) for on ice performance (21, 32, 34, 36, 39, 40, 89). The majority of research has been conducted in adult male and female ice hockey players. For example, Green and coworkers (89) revealed percentage body fat was significantly related to total minutes played in a game in NCAA Division I hockey players. Chiarlitti and coworkers revealed that body composition had a significant influence on several of the NHL combine-specific tests in university male ice hockey players (36).

There is a growing body of work examining younger ice hockey players (80). In our current study, we revealed that body composition was a significant predictor of on-ice performance in male youth ice hockey players. Body mass was correlated with skating speed and agility. The lighter the participant, the more agile and fast they were. Height was not significantly correlated with on-ice performance in our athletes. These findings are supported by Martini and colleagues (80) who examined diverse measures of muscular strength (grip strength), power (seated medicine ball throw), and endurance (chin-up; max repetitions) in elite male and female adolescent (15 years) ice hockey players. The authors revealed that taller and heavier male athletes had better performance in horizontal and vertical jump, grip strength, acceleration, and speed, but not change of direction, aerobic fitness, or upper body power. In comparison, heavier female athletes tended to display poorer performance. The authors concluded that the taller-heavier prototype did not necessarily translate to better on ice performance in elite adolescent hockey players.

### 4.11 Shot velocity

There are considerable anecdotal reports that outline the importance of a hard short (i.e., high shot velocity) with success in ice hockey (at all levels). However, shot velocity is seldom recorded (accurately) during game situations. Moreover, limited research has examined the relationship between off-ice fitness measures and on ice shooting velocity (92). The slapshot of Zdeno Chara (Boston Bruins) during the 2012 NHL All-Star Skills Competition [i.e., 175.5 km·h^−1^ (108.8 mph)] set the benchmark for many, with reports of higher values more recently. Shot velocity, while not an explosive skating movement, does require muscular strength and power in addition to technique (92). Although the latter may be difficult to quantify, we can measure certain attributes that relate to how hard someone can shoot the puck. Bežák and Přidal (92) revealed that upper body muscular strength and power were directly associated with shot velocity (wrist and slap) in adult male professional and semi-professional ice hockey players. Wu and colleagues (93) revealed that player characteristics (including skill level, body size, and strength) were the key factors determining puck velocity during both slap and wrist shots. The authors reported significant relationships between various off-ice measures (including height, weight, 1-repetition maximum bench press, and grip strength) and slap and wrist shots in adult male and female ice hockey players. Different stick stiffness properties did not significantly affect puck velocity in both skilled and less skilled players.

To our knowledge, our study is the first to examine shot velocity and its relationship to off-ice fitness measures in young ice hockey players. In our current study, shot velocity was significantly correlated with body mass, wingspan, one leg squat (muscular strength and balance), Wingate-derived anerobic capacity and fatigue index, and sprinting speed. Sprinting speed (during the 30 m Run Split) was the most relevant variable to predict on-ice shot speed. Our findings compare well to those from older populations (92, 93). From a practical perspective, enhanced body mass, wingspan, anerobic capacity, and muscular strength appear to confer benefits for shot velocity.

### 4.12 Practical applications

Using performance measures, such as those undertaken in the present investigation, are valuable and informative assessments that can be used to assess the physical attributes of athletes. These findings support the utility of a number of off-ice assessments for predicting on-ice performance in ice hockey players under the age of 12. Furthermore, this information provides support for integrating a number of focus areas into athlete off-ice training regimens. For example, emphasizing exercises to improve vertical and horizontal jump performance to enhance on-ice speed and agility. Developing single leg strength and balance through exercises like the single leg squat to improve on-ice performance may also have utility for young ice hockey players. While some assessments such as flexibility may not demonstrate predictive capability for on-ice measures directly, flexibility should not be overlooked for its potential role in injury prevention.

### 4.13 Study limitations and methodological considerations

The purpose of the investigation was to examine the relationships between off-ice assessments and on-ice performance in a sample of male ice hockey players under the age of 12 years. The assessments used in the present investigation have been previously prescribed for an adult elite level athlete; for some participants, this study may have been the first off-ice assessment battery that they have participated in within the context of ice hockey. While many of the findings align with the existing literature on older cohorts of ice hockey players, the next step is to focus on examining both the human and sport-specific physiology underlying these results in relation to age, childhood development, and the sport of ice hockey. Furthermore, future studies with larger sample sizes across different birth years and sexes are warranted. This is especially important for the longitudinal monitoring of player development and to gain a better understanding of the maturational changes that occur with age.

The maximal speed calculations were carried out with only two measurements of time (i.e., the split and total time) for the 30 m run sprint and 54 m skating sprint which may explain why some participants showed small tau times (e.g., ≈ 0.25 s). Therefore, the results of maximal speed and tau times should be interpreted with caution. Further studies should aim to incorporate at least three measurements of time (two split and the total time) to smooth the speed vs. time curve.

## 5 Conclusion

To our knowledge, this investigation was the first to examine the relationships between off-ice assessments and on-ice performance in male ice hockey players under the age of 12 years. Our ultimate goal was to enhance the overall understanding of the varied factors influencing success in youth ice hockey, contributing to the optimization of training regimens and player performance at various levels of the sport. This information provides insight into physical fitness tests that are potentially associated with on-ice performance. We demonstrated that diverse off-ice measures of aerobic fitness, anaerobic power, muscular strength, power, and endurance, and sprinting speed, acceleration, and agility were predictive of on-ice performance. The insights gained from this study can contribute to the refinement of assessment protocols, fostering a comprehensive approach to optimizing player performance and potential. Understanding the connection between objective off-ice testing and on-ice performance can support tailored training programs and player development in male youth ice hockey. Our findings have significant practical considerations for youth ice hockey, particularly considering the increasing demands placed upon ice time in addition to the greater attention and respect afforded to dryland training. From a practical perspective, our study supports coaches and trainers focusing on dryland activities and training that enhance speed, agility, acceleration, and jumping power in the childhood years of ice hockey development.

## Data Availability

The original contributions presented in the study are included in the article/Supplementary Material, further inquiries can be directed to the corresponding author.
