# Supplementary material for: The relationship between off-ice testing and on-ice performance in male youth Ice hockey players
Source: Front Sports Act Living. 2024 Aug 15;6:1418713. doi: 10.3389/fspor.2024.1418713 (PMC11358090; doi:10.3389/fspor.2024.1418713)
Supplement: Supplementary file 1 [file Table1.docx]

**Supplemental material**

The Relationship Between Off-Ice Testing and On-Ice Performance in Male Youth Ice Hockey Players

**Mark S. Rice^1,2†^, Darren E. R. Warburton^3,4†^, Alejandro Gaytan-Gonzalez^3.4^, Veronica K. Jamnik^5^, Kai Kaufman^1,3^, Declan R. D. Warburton^1,3^, Michael Souster^4^, and Shannon S. D. Bredin^1,3*^**

^1^Cognitive and Motor Learning (LEARN) Laboratory, Faculty of Education, University of British Columbia, Vancouver, BC, Canada

^2^School of Kinesiology, University of the Fraser Valley, Chilliwack, BC, Canada

^3^Physical Activity Promotion and Chronic Disease Prevention Unit, Faculty of Education, University of British Columbia, Vancouver, BC, Canada

^4^Experimental Medicine Program, Faculty of Medicine, University of British Columbia, Vancouver, BC, Canada

^5^School of Kinesiology and Health Science, York University, Toronto, ON, Canada

^†^These authors share first authorship.

*** Correspondence:**
Dr. Shannon S. D. Bredin

210-Lower Mall Research Station, 2259 Lower Mall

Faculty of Education, The University of British Columbia

Vancouver, BC, Canada V6T 1Z4
[shannon.bredin@ubc.ca](mailto:shannon.bredin@ubc.ca)

Supplemental Table 1. Correlation between anthropometric, muscular strength, endurance, and power, flexibility, and agility with on-ice performance variables (n = 11).

|  | **15.2 m Sprint**  **time**  **(s)** | **54 m Split (6.1 m) Sprint time (s)** | **54 m Total (54 m) Sprint time (s)** | **15.2 m Sprint speed (m·s^-1^)** | **54 m Split (6.1 m) Sprint speed (m·s^-1^)** | **54 m Total (54 m) Sprint Average speed (m·s^-1^)** | **54 m Total (54 m) Sprint Max speed (m·s^-1^)** | **54 m Sprint tau (s)** | **Agility to the Right (s)** | **Agility to the Left**  **(s)** | **Shot speed (km·h^-1^)** |
| --- | --- | --- | --- | --- | --- | --- | --- | --- | --- | --- | --- |
| Anthropometrics |  |  |  |  |  |  |  |  |  |  |  |
| Weight (kg) | **.68*** | .22 | .29 | **-.68*** | -.17 | -.27 | -.28 | .19 | **.70*** | **.68*** | **.64*** |
| Height (cm) | .35 | -.16 | -.07 | -.35 | .17 | .09 | -.02 | -.18 | .37 | .38 | .47 |
| Wingspan (cm) | .40 | .17 | .26 | -.39 | -.14 | -.24 | -.26 | .14 | .57 | .42 | **.73*** |
| Muscular strength |  |  |  |  |  |  |  |  |  |  |  |
| Grip Strength – Right hand (kg) | -.04 | -.32 | -.27 | .05 | .36 | .28 | .17 | -.30 | .06 | -.03 | .11 |
| Grip Strength – Left hand (kg) | -.18 | -.33 | -.42 | .19 | .39 | .45 | .45 | -.24 | -.01 | -.05 | .21 |
| Grip Strength – Combined (kg) | -.12 | -.36 | -.38 | .13 | .41 | .40 | .35 | -.30 | .03 | -.04 | .18 |
| Muscular endurance |  |  |  |  |  |  |  |  |  |  |  |
| Push-Ups (reps) | -.41 | **-.75**** | **-.67*** | .42 | **.73*** | **.65*** | .42 | **-.74**** | -.48 | -.32 | -.13 |
| Curl-Ups (reps) | .06 | -.43 | -.33 | -.05 | .40 | .32 | .14 | -.45 | .06 | .07 | .04 |
| Muscular power |  |  |  |  |  |  |  |  |  |  |  |
| Standing Long Jump (cm) | **-.66*** | **-.84**** | **-.94***** | **.65*** | **.86***** | **.94***** | **.82**** | **-.74**** | **-.78**** | **-.67*** | -.40 |
| Vertical Jump (cm) | -.50 | **-.83**** | **-.90***** | .48 | **.87***** | **.91***** | **.76**** | **-.74**** | **-.68*** | -.55 | -.37 |
| Single Leg Squat – Right Leg (score) | **-.66*** | -.47 | **-.71*** | **.66*** | .51 | **.72*** | **.81**** | -.32 | **-.64*** | -.58 | -.40 |
| Single Leg Squat – Left Leg  (score) | **-.73*** | -.25 | **-.63*** | **.71*** | .28 | **.64*** | **.88***** | -.08 | **-.72*** | **-.67*** | **-.71*** |
| Single Leg Squat – Combined (score) | **-.73*** | -.36 | **-.70*** | **.72*** | .40 | **.70*** | **.89***** | -.19 | **-.72*** | **-.67*** | **-.61*** |
| Flexibility |  |  |  |  |  |  |  |  |  |  |  |
| Trunk Flexion (cm) | .18 | .19 | .17 | -.16 | -.14 | -.14 | -.08 | .20 | .49 | .38 | .34 |
| Agility |  |  |  |  |  |  |  |  |  |  |  |
| Right – Split (s) | **.64*** | .54 | .53 | **-.64*** | -.57 | -.54 | -.40 | .50 | **.68*** | **.69*** | .09 |
| Right – Total (s) | **.74**** | **.81**** | **.78**** | **-.74**** | **-.83**** | **-.78**** | -.56 | **.77**** | **.75**** | **.71*** | .29 |
| Left – Split (s) | **.69*** | **.82**** | **.88***** | **-.68*** | **-.83**** | **-.87***** | **-.73*** | **.73*** | **.73*** | **.75**** | .25 |
| Left – Total (s) | **.79**** | **.79**** | **.91***** | **-.78**** | **-.82**** | **-.91***** | **-.81**** | **.68*** | **.82**** | **.80**** | .39 |
| Data reported as Pearson correlation coefficients (r). Bold numbers are statistically significant correlations at * p < .05, ** p < .01, *** p < .001 | | | | | | | | | | | |

Supplemental Table 2. Correlation between anaerobic and aerobic fitness with on-ice performance variables (n = 11).

|  | **15.2 m Sprint**  **time**  **(s)** | **54 m Split (6.1 m) Sprint time (s)** | **54 m Total (54 m) Sprint time (s)** | **15.2 m Sprint speed (m·s^-1^)** | **54 m Split (6.1 m) Sprint speed (m·s^-1^)** | **54 m Total (54 m) Sprint Average speed (m·s^-1^)** | **54 m Total (54 m) Sprint Max speed (m·s^-1^)** | **54 m Sprint tau (s)** | **Agility to the Right (s)** | **Agility to the Left**  **(s)** | **Shot speed (km·h^-1^)** |
| --- | --- | --- | --- | --- | --- | --- | --- | --- | --- | --- | --- |
| Anaerobic fitness |  |  |  |  |  |  |  |  |  |  |  |
| Mean Power (W) | .16 | -.45 | -.36 | -.16 | .48 | .38 | .21 | -.44 | .18 | .21 | .15 |
| Peak Power (W) | .54 | -.17 | .04 | -.53 | .19 | -.03 | -.24 | -.24 | .52 | .49 | .56 |
| Minimum Power (W) | -.37 | -.44 | -.53 | .37 | .50 | .55 | .53 | -.35 | -.22 | -.24 | -.10 |
| Anaerobic Capacity (W·kg^-1^) | **-.78**** | **-.62*** | **-.68*** | **.78**** | .60 | **.67*** | .58 | -.56 | **-.75**** | **-.70*** | **-.73*** |
| Anaerobic Power (W·kg^-1^) | -.25 | **-.70*** | -.44 | .26 | **.67*** | .42 | .05 | **-.77**** | -.29 | -.34 | -.04 |
| Fatigue Index (W·s^-1^) | **.77**** | .05 | .32 | **-.77**** | -.06 | -.32 | -.51 | -.06 | **.69*** | **.67*** | .60 |
| Fatigue Index (%) | **.75**** | .11 | .39 | **-.75**** | -.13 | -.39 | -.59 | -.02 | **.61*** | .58 | **.71*** |
| 30 m Run – Split (6.1 m) Sprint time (s) | **.90***** | .26 | .57 | **-.89***** | -.26 | -.56 | **-.73*** | .13 | **.85***** | **.86***** | **.77**** |
| 30 m Run – Total (30 m) Sprint time (s) | **.86***** | **.82**** | **.94***** | **-.85***** | **-.82**** | **-.93***** | **-.83**** | **.71*** | **.91***** | **.86***** | .52 |
| 30 m Run – Split (6.1 m) Sprint speed (m·s^-1^) | **-.88***** | -.24 | -.54 | **.88***** | .24 | .52 | **.70*** | -.11 | **-.82**** | **-.83**** | **-.79**** |
| 30 m Run – Total (30 m) Average Sprint speed (m·s^-1^) | **-.86***** | **-.82**** | **-.94***** | **.86***** | **.83**** | **.94***** | **.84**** | **-.71*** | **-.89***** | **-.85**** | -.49 |
| 30 m Run – Total (30 m) Max Sprint speed (m·s^-1^) | **-.73*** | **-.90***** | **-.95***** | **.73*** | **.91***** | **.95***** | **.77**** | **-.82**** | **-.78**** | **-.72*** | -.33 |
| 30 m Run Sprint tau (s) | .31 | -.56 | -.26 | -.30 | .57 | .27 | -.09 | **-.64*** | .19 | .27 | .50 |
| Aerobic fitness |  |  |  |  |  |  |  |  |  |  |  |
| Leger Stage Completed | **-.73*** | **-.84**** | **-.88***** | **.73*** | **.85***** | **.87***** | **.71*** | **-.77**** | **-.82**** | **-.72*** | -.42 |
| VO_2_max (ml·kg^-1^·min^-1^) | **-.77**** | **-.76**** | **-.79**** | **.78**** | **.76**** | **.78**** | **.62*** | **-.70*** | **-.84**** | **-.78**** | -.46 |
| Data reported as Pearson correlation coefficients (r). Bold numbers are statistically significant correlations at * p < .05, ** p < .01, *** p < .001 | | | | | | | | | | | |

Supplemental Table 3. Regression coefficients for predicting on-ice 15.2 m sprint time and speed (n = 11).

|  | **Time (s)** | | | | **Speed (m·s^-1^)** | | | |
| --- | --- | --- | --- | --- | --- | --- | --- | --- |
| **Variable** | **Single variable models^1^** | | | | **Single variable models^1^** | | | |
|  | **Intercept** | **β** | **p** | **SEE** | **Intercept** | **β** | **p** | **SEE** |
| Weight (kg) | 1.675 | 0.009 | 0.021 | 0.07 | 8.84 | -0.04 | 0.021 | 0.25 |
| Standing Long Jump (cm) | 2.513 | -0.003 | 0.026 | 0.07 | 5.71 | 0.01 | 0.030 | 0.26 |
| Single Leg Squat – Right Leg (score) | 2.290 | -0.005 | 0.027 | 0.07 | 6.52 | 0.02 | 0.027 | 0.26 |
| Single Leg Squat – Left Leg (score) | 2.240 | -0.004 | 0.011 | 0.06 | 6.73 | 0.02 | 0.015 | 0.24 |
| Single Leg Squat – Combined (score) | 2.287 | -0.003 | 0.011 | 0.06 | 6.54 | 0.01 | 0.013 | 0.24 |
| Agility Right – Split (s) | 1.002 | 0.328 | 0.032 | 0.07 | 11.39 | -1.24 | 0.033 | 0.26 |
| Agility Right – Total (s) | 0.803 | 0.205 | 0.009 | 0.06 | 12.18 | -0.78 | 0.009 | 0.23 |
| Agility Left – Split (s) | 1.070 | 0.313 | 0.019 | 0.07 | 11.09 | -1.17 | 0.021 | 0.25 |
| Agility Left – Total (s) | 0.707 | 0.223 | 0.004 | 0.06 | 12.46 | -0.84 | 0.004 | 0.21 |
| Anaerobic Capacity (W·kg^-1^) | 2.528 | -0.080 | 0.005 | 0.06 | 5.61 | 0.30 | 0.005 | 0.21 |
| Fatigue index (W·s^-1^) | 1.800 | 0.041 | 0.006 | 0.06 | 8.37 | -0.16 | 0.006 | 0.22 |
| Fatigue index (%) | 1.752 | 0.006 | 0.008 | 0.06 | 8.55 | -0.02 | 0.008 | 0.22 |
| 30 m Run – Split (6.1 m) Sprint time (s) | 0.913 | .741 | < 0.001 | 0.04 | 11.67 | -2.77 | < 0.001 | 0.16 |
| 30 m Run – Total (30 m) Sprint time (s) | 0.962 | 0.191 | < 0.001 | 0.05 | 11.51 | -0.72 | < 0.001 | 0.18 |
| 30 m Run – Split (6.1 m) Sprint speed (m·s^-1^) | 3.099 | -0.263 | < 0.001 | 0.04 | 3.50 | 0.99 | < 0.001 | 0.16 |
| 30 m Run – Total (30 m) Sprint Average speed (m·s^-1^) | 3.075 | -0.194 | < 0.001 | 0.05 | 3.57 | 0.73 | < 0.001 | 0.18 |
| 30 m Run – Total (30 m) Sprint Max speed (m·s^-1^) | 2.768 | -0.126 | 0.011 | 0.06 | 4.72 | 0.47 | 0.012 | 0.23 |
| Leger Stage Completed | 2.258 | -0.035 | 0.010 | 0.06 | 6.63 | 0.13 | 0.010 | 0.23 |
| VO_2_max (ml·kg^-1^·min^-1^) | 2.816 | -0.015 | 0.005 | 0.06 | 4.51 | 0.06 | 0.005 | 0.21 |
| ^1^ Each variable was individually tested to predict on-ice 15.2 m sprint time or speed.  SEE = Standard error of the estimate (or the prediction). | | | | | | | | |

Supplemental Table 4. Regression coefficients for predicting on-ice 54 m split (6.1 m) sprint time and speed (n = 11).

|  | **Time (s)** | | | | **Speed (m·s^-1^)** | | | |
| --- | --- | --- | --- | --- | --- | --- | --- | --- |
| **Variable** | **Single variable models^1^** | | | | **Single variable models^1^** | | | |
|  | **Intercept** | **β** | **p** | **SEE** | **Intercept** | **β** | **p** | **SEE** |
| Push-Ups (reps) | 1.921 | -0.038 | 0.008 | 0.13 | 3.12 | 0.09 | 0.011 | 0.33 |
| Standing Long Jump (cm) | 2.944 | -0.009 | 0.001 | 0.11 | 0.49 | 0.02 | < 0.001 | 0.24 |
| Vertical Jump (cm) | 2.325 | -0.021 | 0.001 | 0.11 | 2.03 | 0.05 | < 0.001 | 0.24 |
| Agility Right – Total (s) | -1.363 | 0.495 | 0.002 | 0.12 | 11.20 | -1.23 | 0.002 | 0.27 |
| Agility Left – Split (s) | -0.885 | 0.811 | 0.002 | 0.11 | 10.01 | -2.01 | 0.002 | 0.27 |
| Agility Left – Total (s) | -1.302 | 0.488 | 0.004 | 0.12 | 11.16 | -1.23 | 0.002 | 0.28 |
| Anaerobic Capacity (W·kg^-1^)^2^ | 2.453 | -0.140 | 0.042 | 0.15 | - | - | - | - |
| Anaerobic Power (W·kg^-1^) | 3.030 | -0.168 | 0.016 | 0.14 | 0.55 | 0.39 | 0.025 | 0.36 |
| 30 m Run – Total (30 m) Sprint time (s) | -0.636 | 0.397 | 0.002 | 0.11 | 9.36 | -0.98 | 0.002 | 0.27 |
| 30 m Run – Total (30 m) Sprint Average speed (m·s^-1^) | 3.758 | -0.403 | 0.002 | 0.11 | -1.47 | 1.00 | 0.002 | 0.27 |
| 30 m Run – Total (30 m) Sprint Max speed (m·s^-1^) | 3.599 | -0.340 | < 0.001 | 0.09 | -1.09 | 0.84 | < 0.001 | 0.20 |
| Leger Stage Completed | 2.176 | -0.089 | 0.001 | 0.11 | 2.45 | 0.22 | < 0.001 | 0.25 |
| VO_2_max (ml·kg^-1^·min^-1^) | 3.293 | -0.33 | 0.006 | 0.13 | -0.25 | 0.08 | 0.006 | 0.31 |
| ^1^ Each variable was individually tested to predict on-ice 54 m split (6.1 m) sprint time or speed.  ^2^ Statistically significantly correlated with sprint time but not sprint speed.  SEE = Standard error of the estimate (or the prediction). | | | | | | | | |

Supplemental Table 5. Regression coefficients for predicting on-ice 54 m total (54 m) sprint time and average speed (n = 11).

|  | **Time (s)** | | | | **Speed (m·s^-1^)** | | | |
| --- | --- | --- | --- | --- | --- | --- | --- | --- |
| **Variable** | **Single variable models^1^** | | | | **Single variable models^1^** | | | |
|  | **Intercept** | **β** | **p** | **SEE** | **Intercept** | **β** | **p** | **SEE** |
| Push-Ups (reps) | 9.59 | -0.09 | 0.025 | 0.36 | 5.61 | 0.06 | 0.029 | 0.26 |
| Standing Long Jump (cm) | 12.66 | -0.02 | < 0.001 | 0.18 | 3.43 | 0.02 | < 0.001 | 0.12 |
| Vertical Jump (cm) | 10.86 | -0.06 | < 0.001 | 0.22 | 4.69 | 0.04 | < 0.001 | 0.15 |
| Single Leg Squat – Right Leg (score) | 10.41 | -0.03 | 0.015 | 0.34 | 5.00 | 0.02 | 0.013 | 0.24 |
| Single Leg Squat – Left Leg (score) | 9.86 | -0.02 | 0.036 | 0.38 | 5.40 | 0.01 | 0.035 | 0.26 |
| Single Leg Squat – Combined (score) | 10.20 | -0.01 | 0.017 | 0.35 | 5.16 | 0.01 | 0.016 | 0.24 |
| Agility Right – Total (s) | 1.85 | 1.18 | 0.005 | 0.31 | 11.04 | -0.83 | 0.005 | 0.22 |
| Agility Left – Split (s) | 2.25 | 2.18 | < 0.001 | 0.23 | 10.72 | -1.52 | < 0.001 | 0.17 |
| Agility Left – Total (s) | 0.63 | 1.40 | < 0.001 | 0.20 | 11.91 | -0.98 | < 0.001 | 0.14 |
| Anaerobic Capacity (W·kg^-1^) | 11.26 | -0.38 | 0.020 | 0.36 | 4.46 | 0.27 | 0.024 | 0.25 |
| 30 m Run – Total (30 m) Sprint time (s) | 2.53 | 1.14 | < 0.001 | 0.17 | 10.52 | -0.79 | < 0.001 | 0.12 |
| 30 m Run – Total (30 m) Sprint Average speed (m·s^-1^) | 15.11 | -1.15 | < 0.001 | 0.17 | 1.74 | 0.81 | < 0.001 | 0.12 |
| 30 m Run – Total (30 m) Sprint Max speed (m·s^-1^) | 14.15 | -0.89 | < 0.001 | 0.16 | 2.40 | 0.63 | < 0.001 | 0.11 |
| Leger Stage Completed | 10.40 | -0.23 | < 0.001 | 0.23 | 5.03 | 0.16 | < 0.001 | 0.17 |
| VO_2_max (ml·kg^-1^·min^-1^) | 13.25 | -0.08 | 0.004 | 0.30 | 3.07 | 0.06 | 0.005 | 0.22 |
| ^1^ Each variable was individually tested to predict on-ice 54 m total (54 m) sprint time or average speed.  SEE = Standard error of the estimate (or the prediction). | | | | | | | | |

Supplemental Table 6. Regression coefficients for predicting on-ice 54 m total (54 m) sprint max speed (n = 11).

|  | **Speed (m·s^-1^)** | | | |
| --- | --- | --- | --- | --- |
| **Variable** | **Single variable models^1^** | | | |
|  | **Intercept** | **β** | **p** | **SEE** |
| Standing Long Jump (cm) | 4.83 | 0.01 | 0.002 | 0.16 |
| Vertical Jump (cm) | 5.75 | 0.03 | 0.007 | 0.18 |
| Single Leg Squat – Right Leg (score) | 5.68 | 0.02 | 0.003 | 0.16 |
| Single Leg Squat – Left Leg (score) | 5.88 | 0.02 | < 0.001 | 0.13 |
| Single Leg Squat – Combined (score) | 5.70 | 0.01 | < 0.001 | 0.13 |
| Agility Left – Split (s) | 9.73 | -1.00 | 0.012 | 0.19 |
| Agility Left – Total (s) | 10.80 | -0.70 | 0.002 | 0.16 |
| 30 m Run – Split (6.1 m) Sprint time (s) | 9.43 | -1.82 | 0.011 | 0.19 |
| 30 m Run – Total (30 m) Sprint time (s) | 9.82 | -0.56 | 0.001 | 0.15 |
| 30 m Run – Split (6.1 m) Sprint speed (m·s^-1^) | 4.13 | 0.63 | 0.017 | 0.20 |
| 30 m Run – Total (30 m) Sprint Average speed (m·s^-1^) | 3.57 | 0.58 | 0.001 | 0.15 |
| 30 m Run – Total (30 m) Sprint Max speed (m·s^-1^) | 4.29 | 0.41 | 0.005 | 0.17 |
| Leger Stage Completed | 6.00 | 0.10 | 0.015 | 0.19 |
| VO_2_max (ml·kg^-1^·min^-1^) | 4.77 | 0.04 | 0.042 | 0.21 |
| ^1^ Each variable was individually tested to predict on-ice 54 m total (54 m) sprint max speed.  SEE = Standard error of the estimate (or the prediction). | | | | |

Supplemental Table 7. Regression coefficients for predicting on-ice 54 m sprint tau (n = 11).

|  | **Tau (s)** | | | |
| --- | --- | --- | --- | --- |
| **Variable** | **Single variable models^1^** | | | |
|  | **Intercept** | **β** | **p** | **SEE** |
| Push-Ups (reps) | 1.226 | -0.050 | 0.009 | 0.17 |
| Standing Long Jump (cm) | 2.361 | -0.010 | 0.010 | 0.18 |
| Vertical Jump (cm) | 1.655 | -0.025 | 0.009 | 0.17 |
| Agility Right – Total (s) | -2.896 | 0.619 | 0.006 | 0.17 |
| Agility Left – Split (s) | -2.152 | 0.963 | 0.010 | 0.18 |
| Agility Left – Total (s) | -2.524 | 0.559 | 0.020 | 0.19 |
| Anaerobic Power (W·kg^-1^) | 2.898 | -0.244 | 0.005 | 0.17 |
| 30 m Run – Total (30 m) Sprint time (s) | -1.783 | 0.459 | 0.014 | 0.18 |
| 30 m Run – Total (30 m) Sprint Average speed (m·s^-1^) | 3.282 | -0.464 | 0.014 | 0.18 |
| 30 m Run – Total (30 m) Sprint Max speed (m·s^-1^) | 3.201 | -0.408 | 0.002 | 0.15 |
| 30 m Run Sprint tau (s) | 1.438 | -1.464 | 0.035 | 0.20 |
| Leger Stage Completed | 1.497 | -0.107 | 0.006 | 0.17 |
| VO_2_max (ml·kg^-1^·min^-1^) | 2.858 | -0.039 | 0.016 | 0.19 |
| ^1^ Each variable was individually tested to predict on-ice 54 m sprint tau.  SEE = Standard error of the estimate (or the prediction). | | | | |

Supplemental Table 8. Regression coefficients for predicting on-ice agility to the right time (n = 11).

|  | **Time (s)** | | | |
| --- | --- | --- | --- | --- |
| **Variable** | **Single variable models^1^** | | | |
|  | **Intercept** | **β** | **p** | **SEE** |
| Weight (kg) | 8.83 | 0.04 | 0.016 | 0.30 |
| Standing Long Jump (cm) | 13.20 | -0.02 | 0.005 | 0.26 |
| Vertical jump (cm) | 11.79 | -0.04 | 0.022 | 0.31 |
| Single Leg Squat – Right Leg (score) | 11.70 | -0.02 | 0.035 | 0.32 |
| Single Leg Squat – Left Leg (score) | 11.51 | -0.02 | 0.012 | 0.29 |
| Single Leg Squat – Combined (score) | 11.71 | -0.01 | 0.013 | 0.29 |
| Agility Right – Split (s) | 5.45 | 1.62 | 0.021 | 0.31 |
| Agility Right – Total (s) | 4.69 | 0.98 | 0.007 | 0.28 |
| Agility Left – Split (s) | 5.78 | 1.55 | 0.010 | 0.28 |
| Agility Left – Total (s) | 4.17 | 1.08 | 0.002 | 0.24 |
| Anaerobic Capacity (W·kg^-1^) | 12.77 | -0.36 | 0.008 | 0.28 |
| Fatigue index (W·s^-1^) | 9.56 | 0.17 | 0.019 | 0.30 |
| Fatigue index (%) | 9.45 | 0.02 | 0.044 | 0.33 |
| 30 m Run – Split (6.1 m) Sprint time (s) | 5.61 | 3.26 | < 0.001 | 0.22 |
| 30 m Run – Total (30 m) Sprint time (s) | 5.28 | 0.94 | < 0.001 | 0.18 |
| 30 m Run – Split (6.1 m) Sprint speed (m·s^-1^) | 15.16 | -1.14 | 0.002 | 0.24 |
| 30 m Run – Total (30 m) Sprint Average speed (m·s^-1^) | 15.58 | -0.94 | < 0.001 | 0.19 |
| 30 m Run – Total (30 m) Sprint Max speed (m·s^-1^) | 14.24 | -0.63 | 0.004 | 0.26 |
| Leger Stage Completed | 11.75 | -0.19 | 0.002 | 0.24 |
| VO_2_max (ml·kg^-1^·min^-1^) | 14.53 | -0.08 | 0.001 | 0.23 |
| ^1^ Each variable was individually tested to predict on-ice agility to the right time.  SEE = Standard error of the estimate (or the prediction). | | | | |

Supplemental Table 9. Regression models for predicting on-ice agility to the left time (n = 11).

|  | **Time (s)** | | | |
| --- | --- | --- | --- | --- |
| **Variable** | **Single variable models^1^** | | | |
|  | **Intercept** | **β** | **p** | **SEE** |
| Weight (kg) | 8.88 | 0.05 | 0.022 | 0.36 |
| Standing Long Jump (cm) | 13.50 | -0.02 | 0.024 | 0.37 |
| Single Leg Squat – Left Leg (score) | 11.88 | -0.02 | 0.023 | 0.36 |
| Single Leg Squat – Combined (score) | 12.09 | -0.01 | 0.026 | 0.37 |
| Agility Right – Split (s) | 4.77 | 1.93 | 0.019 | 0.36 |
| Agility Right – Total (s) | 4.34 | 1.08 | 0.014 | 0.35 |
| Agility Left – Split (s) | 5.07 | 1.88 | 0.007 | 0.32 |
| Agility Left – Total (s) | 3.50 | 1.24 | 0.003 | 0.30 |
| Anaerobic Capacity (W·kg^-1^) | 13.27 | -0.40 | 0.017 | 0.35 |
| Fatigue index (W·s^-1^) | 9.69 | 0.20 | 0.023 | 0.36 |
| 30 m Run – Split (6.1 m) Sprint time (s) | 4.93 | 3.90 | < 0.001 | 0.25 |
| 30 m Run – Total (30 m) Sprint time (s) | 4.96 | 1.05 | < 0.001 | 0.25 |
| 30 m Run – Split (6.1 m) Sprint speed (m·s^-1^) | 16.32 | -1.36 | 0.001 | 0.27 |
| 30 m Run – Total (30 m) Sprint Average speed (m·s^-1^) | 16.45 | -1.05 | 0.001 | 0.26 |
| 30 m Run – Total (30 m) Sprint Max speed (m·s^-1^) | 14.82 | -0.68 | 0.013 | 0.34 |
| Leger Stage Completed | 12.05 | -0.19 | 0.012 | 0.34 |
| VO_2_max (ml·kg^-1^·min^-1^) | 15.16 | -0.08 | 0.005 | 0.31 |
| ^1^ Each variable was individually tested to predict on-ice agility to the left time.  SEE = Standard error of the estimate (or the prediction). | | | | |

Supplemental Table 10. Regression coefficients for predicting on-ice shot speed (n = 11).

|  | **Speed (km∙h^-1^)** | | | |
| --- | --- | --- | --- | --- |
| **Variable** | **Single variable models^1^** | | | |
|  | **Intercept** | **β** | **p** | **SEE** |
| Weight (kg) | 38.1 | 0.65 | .033 | 5.2 |
| Wingspan (cm) | -26.8 | 0.63 | .010 | 4.6 |
| Single Leg Squat – Left Leg (score) | 78.9 | -0.31 | .014 | 4.7 |
| Single Leg Squat – Combined (score) | 79.1 | -0.16 | .047 | 5.3 |
| Anaerobic Capacity (W·kg^-1^) | 98.7 | -5.68 | .010 | 4.6 |
| Fatigue index (%) | 43.7 | 0.41 | .015 | 4.8 |
| 30 m Run – Split (6.1 m) Sprint time (s) | -8.9 | 47.8 | .005 | 4.3 |
| 30 m Run – Split (6.1 m) Sprint speed (m·s^-1^) | 134.7 | -17.6 | .004 | 4.1 |
| ^1^ Each variable was individually tested to predict on-ice shot speed.  SEE = Standard error of the estimate (or the prediction). | | | | |
